# EPH receptor A2 governs a feedback loop that activates Wnt/β-catenin signaling in gastric cancer

**DOI:** 10.1038/s41419-018-1164-y

**Published:** 2018-11-19

**Authors:** Qiu Peng, Ling Chen, Wei Wu, Jia Wang, Xiang Zheng, Zihua Chen, Qin Jiang, Jiaqi Han, Lingyu Wei, Lujuan Wang, Jin Huang, Jian Ma

**Affiliations:** 1 Department of Oncology, Xiangya Hospital, Central South University, Changsha, Hunan China; 2 Cancer Research Institute, School of Basic Medical Science, Central South University, Hunan, China; 3 Department of Gastrointestinal Surgery, Xiangya Hospital, Central South University, Hunan, China; 4 Hunan Key Laboratory of Nonresolving Inflammation and Cancer, Key Laboratory of Carcinogenesis of the Chinese Ministry of Health, Key Laboratory of Carcinogenesis and Cancer Invasion of the Chinese Ministry of Education, Hunan Key Laboratory of Translational Radiation Oncology at Hunan Cancer Hospital, Changsha, Hunan China

## Abstract

The erythropoietin-producing hepatoma (EPH) receptor A2 (EphA2) belongs to the Eph family of receptor tyrosine kinases. EphA2 is highly correlated with the formation of many solid tumors and has been linked to the dysregulation of signaling pathways that promote tumor cell proliferation, migration, and invasion as well as angiogenesis. Deregulation of Wnt signaling is implicated in many forms of human disease including gastric cancer. We previously reported that EphA2 promotes the epithelial–mesenchymal transition through Wnt/β-catenin signaling in gastric cancer. Herein, we present a novel mechanism by which EphA2 regulates Wnt/β-catenin signaling. EphA2 acts as a receptor for Wnt ligands and recruits Axin1 to the plasma membrane by directly binding Dvl2. The EphA2-Dvl2/Axin1 interaction was enhanced by Wnt3a treatment, suggesting that EphA2 acts as a functional receptor for the Wnt/β-catenin pathway and plays a vital role in downstream signaling. We showed that Dvl2 mediates the EphA2-Axin1 interaction by binding to the tyrosine kinase domain of EphA2. We propose that EphA2/Dvl2/Axin1 forms a complex that destabilizes the β-catenin destruction complex and allows β-catenin to translocate to the nucleus and initiate the transcription of *c-MYC*, the primary Wnt signaling target gene. Intriguingly, c-MYC could bind directly to the *EphA2* and *Wnt1* promoter to enhance their transcription. The entire process formed an EphA2-mediated feed-forward loop. A small molecular inhibitor of EphA2 potently inhibited the proliferation of gastric cancer in vitro and in vivo, including gastric cancer patient–derived xenografts. Thus, our data identify EphA2 as an excellent candidate for gastric cancer therapy.

## Introduction

The erythropoietin-producing hepatoma (EPH) family is the largest family of receptor tyrosine kinases, the dysfunction of which is recognized as a key initiator of carcinogenesis^1^. Members of this family enhance or suppress tumor development depending on their mode of activation. For instance, ligand-dependent signaling induced by EphrinA1, a ligand of the EPH receptor A2 (EphA2), is tumor suppressive. In contrast, EphA2 can be activated by interaction with other cell-surface molecules in cancer cells, thus amplifying MAPK, RAS, and AKT signalings, which enhances tumor development^2,3^. EphA2 overexpression has been observed in a wide variety of neoplasms such as gastric cancer, colorectal cancer, etc. EphA2 acts as a key driver of metastasis and is a predictor of poor prognosis in various cancers^4–7^. We previously reported that EphA2 overexpression is associated with poor prognosis for gastric cancer patients and promotes the epithelial-mesenchymal transition (EMT) through the Wnt/β-catenin pathway in gastric cancer cells^8–11^. However, the exact mechanisms of the EphA2 regulation of Wnt/β-catenin signaling in gastric cancer is unclear.

Wnt signaling is an evolutionarily conserved pathway that controls cell-to-cell interactions during embryogenesis and contributes to tissue homeostasis in most of the organ systems in adult^12^. Moreover, the Wnt pathway plays a key role in the proliferation, differentiation, development and maintenance of cancer stem cells^13^. Dysregulation of Wnt signaling is implicated in many forms of human disease including gastric cancer^14^. When the Wnt pathway is “on”, Wnt ligands bind to the Frizzled receptor and LRP5/6 receptors, which convey the signal to intracellular components that then promote the recruitment of Disheveled (Dvl) to the plasma membrane. In turn, Dvl further recruits Axin1 to the membrane and forms a complex with Frizzled and LRP5/6. Finally, the β-catenin destruction complexes are disassembled, which allows unphosphorylated β-catenin to accumulate and subsequently translocate to the nucleus^15,16^.

In this study, we discovered a novel mechanism by which EphA2 drives a feed-forward loop that regulates Wnt signaling; and targeting EphA2 significantly inhibits the proliferation of gastric cancer in vitro and in vivo.

**Fig. 1:**
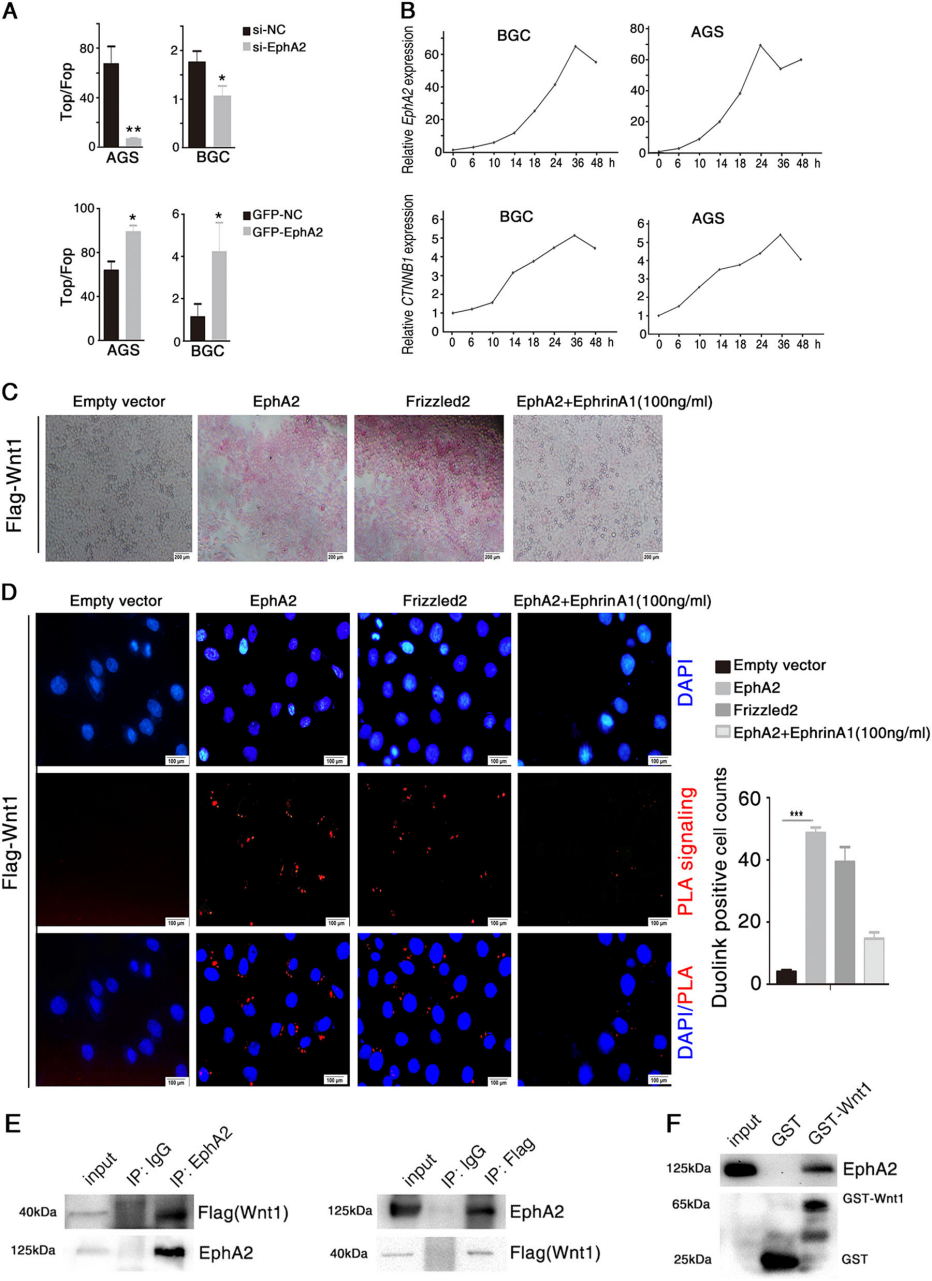
EphA2 interacts with Wnt1. **a** AGS, and BGC823 cells were transfected for 48 h with an siRNA targeting EphA2 (upper panel) or with an EphA2-expressing vector (lower panel). Wnt/β-catenin signaling activity assayed by TOP-flash/FOP-flash luciferase reporter assay. NC: negative control (vector). **b** Dynamic changes of *EphA2* and *CTNNB1* expression levels in BGC823 and AGS cells transfected with an EphA2-expressing vector for the indicated times as assessed with real-time qPCR. **c**, **d** pcDNA3.1-EphA2 was transfected into HEK293 cells for 48 h and incubated with Flag-Wnt1 conditional medium (CM) for an additional 3 h. Frizzled2-expressing vector were used as a positive control, whereas an empty vector was used as a negative control. 100 ng/ml EphrinA1 (the ligand of EphA2) was used to block EphA2 function. **c** Wnt1 binds EphA2 at the cell surface. Staining was performed as described in the Methods. **d** Proximity ligation assays (PLA) for EphA2 and Wnt1 interaction as described in the Methods. **e** pcDNA3.1-EphA2 and Flag-Wnt1 were co-expressed in HEK293 cells for 48 h, cell lysate was subjected to co-IP assay with anti-EphA2 (left) or anti-Flag (right) antibodies. **f** GST pull-down assayed the direct interaction between Wnt1 and EphA2. After sedimentation with Glutathione-beads, Western blot examined the pulled down proteins by using anti-EphA2. Significant differences were determined with the Student’s *t*-test. *P < 0.05, **P < 0.01, ***P < 0.001 compared with control group

## Materials and methods

### Antibodies and reagents

Antibodies to proteins were obtained from the following sources: Flag (#F1804): Sigma-Aldrich; Wnt1 (#sc-5630) and ubiquitin (#sc-9133): Santa Cruz Biotechnology; EphA2(#6997), GST (glutathione S-transferase; #2622), Dvl2 (#3224), c-Myc (#13887), Axin1 (#2087), β-catenin (#8480), histone H3 (#4499), phos-β-catenin (#9565), GSK3β (#9315), and β-TRCP (#4394): Cell Signaling Technology; HA (hemagglutinin; #TA100012): Origene; GAPDH (#D190090): Sangon (Shanghai, China); α-tubulin (#66031-1-Ig): Proteintech. Reagent sources were as follows: recombinant human proteins Wnt3a (#5036-WN) and EphrinA1 (#6417-A1): R&D Systems; EphA2 inhibitor ALW-II-41-27(ALW): MedChem Express; MG132 proteasome inhibitor: Selleck; Dual-Luciferase Reporter Assay System: Promega.

### Cell culture, plasmid construction, and transfection with short interfering RNA (siRNA)

The human gastric cancer cell lines AGS and BGC823 were cultured in RPMI-1640 medium supplemented with 10% fetal calf serum (FBS). HEK293 cells were cultured in Dulbecco’s modified Eagle medium (Gibco) with 1 g/l glucose and 10% FBS. All cell lines were obtained from ATCC. The cell lines tested negative for mycoplasma contamination. All cell lines were used for experiment within 3 months of thawing. All cell lines were authenticated by short tandem repeat profiling prior to use. DNA fragments encoding Flag-EphA2, GFP-EphA2, VN-EphA2, VC-Axin1, VC-Dvl2, RFP-Dvl2, RFP-Wnt1, RFP-Axin1, HA-c-MYC, and Flag-Wnt1 were generated by PCR and cloned into a Flag-tagged (p3xFLAG-CMV-10) or GFP-tagged (pEGFP-N1) empty vector. Different constructs for N-terminal–truncated EphA2 sequences were generated by PCR and cloned into Flag-tagged empty vectors and verified by sequencing. siRNAs were purchased from Ribobio (Guangzhou, China). Plasmids and siRNAs were transfected into cells using Lipofectamine 3000 (Invitrogen). Stable EphA2 short hairpin RNA (shRNA) knockdown lines were generated by infecting AGS and BGC823 cells with three types of reagents: lentiviruses harboring pGLV3/H1/GFP + Puro vector (Genepharma, China), vectors harboring human EphA2 shRNA targeting sequences (#1: 5′-GATGAAAGCCATCAATGATGG-3′; #2: 5′-GCCATTTCCTACCGGAAGTTC-3′; #3: 5′-GCTCAAGTTTACTGTACGTGA-3′), or control GFP-targeting sequences (5′-TTCTCCGAACGTGTCACGT-3′). Selection was with puromycin (2–3 μg/ml). EphA2 knockdown was confirmed by western blotting.

### Real-time quantitative PCR (qPCR)

cDNA was synthesized from 2 μg total RNA with a reverse transcription kit (Promega). mRNA level was evaluated using SYBR Green real-time qPCR (Takara). Human *GAPDH* was amplified in parallel as an internal control. Expression of each gene was quantified by measuring cycle threshold values, and the 2^–ΔΔCt^ method was used to calculate relative changes in gene expression. Primers are listed in Supplementary Table S1.

### Western blotting

Extracts of cells lysed with RIPA buffer were cleared by centrifugation. Lysates (50 μg of protein) were subjected to SDS-PAGE, and the separated bands were transferred to a polyvinylidene difluoride membrane (Millipore) that was then probed with various antibodies.

### Subcellular fractionation

Cytoplasmic and nuclear fractions were isolated with nuclear and cytoplasmic protein extraction kits (Beyotime, China).

### Immunohistochemistry

Paraffin-embedded sections were cut 4 µm thick, then deparaffinized and rehydrated. EphA2, c-Myc, β-catenin, Ki67, and CD31 were detected by immunohistochemical staining as described^17^.

### Co-immunoprecipitation (co-IP)

Co-IP assays were performed as described^18^. Briefly, cell lysates were incubated with 2 μg antibody at 4 °C overnight. Protein A/G-Sepharose beads (Millipore) were added, and the mix was incubated for 2 h at 4 °C. The immunocomplexes were subsequently washed with lysis buffer three times and subjected to SDS-PAGE.

### Glutathione S-transferase (GST) pull-down assay

GST pull-down assays were performed as described^19^. Briefly, recombinant proteins were expressed as GST fusions in *Escherichia coli* BL21. Cells were lysed in phosphate-buffered saline (PBS) containing 1% Triton X-100 and inhibitors of proteases and phosphatases. Lysates containing GST fusion proteins were mixed with glutathione-coupled beads for 2 h at 4 °C, and the beads were washed with PBS three times. Proteins were added to the glutathione-beads and incubated overnight at 4 °C. Beads were washed with PBS three times, resolved by SDS-PAGE, and subjected to western blotting and Coomassie staining.

### Chromatin immunoprecipitation (ChIP)

ChIP assays were performed as described^20^. Briefly, AGS cells were treated with 1% formaldehyde and incubated for 10 min to generate DNA-protein crosslinks. Cell lysates were then sonicated to produce chromatin fragments of 200–300 bp and immunoprecipitated with anti-c-Myc or with IgG (control). Antibody-bound complexes were precipitated with Protein A/G-Sepharose beads. The DNA fragments in the immunoprecipitated complexes were released by reversing the crosslinks at 65 °C for 5 h, and purified DNA was analyzed by PCR and agarose gel electrophoresis. PCR was performed using promoter-specific primers for *EphA2* or *Wnt1* with amplification of the c-MYC-binding regions. Primers are listed in Supplementary Table S2.

### Cell proliferation assay, migration and invasion assay, and plated colony formation assay

Cell proliferation assays were carried out as described^21^ using the Cell Counting Kit-8 (CCK-8; Biotool, China). The migration and invasion assay was as described^21^. Briefly, cells (1 × 10^5^) were seeded onto the upper chamber in 200 μl serum-free RPMI-1640; the lower compartment was filled with 500 μl RPMI-1640 supplemented with 10% FBS. After 24 h of incubation, migrated and invasive cells on the lower surface of the filter were fixed and stained using crystal violet. Cells on the upper side were removed using a rubber scraper. Data represent counts of migrated and invasive cells. Experiments were performed in triplicate. For the colony formation assay, cells (1 × 10^3^/ml per well) were seeded in 6-well plates and cultured for 14 days in RPMI-1640 supplemented with 10% FBS. Colonies were fixed with methanol and stained with crystal violet, then scored using Image J software.

### Flow cytometry

A cell-cycle and apoptosis analysis kit with propidium iodide staining reagent (Beyotime) was used for flow cytometric analysis. Cells were harvested by trypsinization, washed once with cold PBS, and suspended in 70% ethanol; cells were fixed by paraformaldehyde overnight with rotation. Before staining, the cells were washed with PBS. Then, cells were incubated with staining buffer (Beyotime) containing propidium iodide and RNase A in a 37 °C water bath for 0.5 h and then analyzed with flow cytometry.

### Immunofluorescence

Cells were fixed in medium containing 3.7% paraformaldehyde for 1 h and then permeabilized using 0.5% Triton X-100 and blocked using normal goat serum. The primary antibodies were added and incubated at room temperature for 2 h. Alexa Fluor 488-conjugated or 568-conjugated secondary antibodies (Beyotime) were added and incubated for 1 h. Stained cells were examined using a fluorescence microscope.

### TUNEL assay

Terminal deoxynucleotidyl transferase-mediated dUTP nick end labeling (TUNEL) assay was performed using DeadEnd Fluorometric TUNEL System (Promega) according to the manufacturer’s recommended protocol.

### TOP-flash/FOP-flash luciferase reporter assay

Cells were serum-starved overnight and co-transfected with 200 ng TOP-flash or FOP-flash expression plasmid and 50 ng pRL-TK using Lipofectamine 3000. The activities of both firefly and Renilla luciferase reporters were determined at 48 h post-transfection using the Dual Luciferase Assay kit (Promega). The TOP-flash reporter activity is presented as the relative ratio of firefly-to-Renilla luciferase activities, and the TOP/FOP ratio was used as a measure of β-catenin–driven transcription^22^.

### Gene set enrichment analysis (GSEA)

We divided primary gastric adenocarcinoma specimens^23^ into two groups based on *EphA2* expression; specimens in the top 30% were designated as EphA2^high^ and in the bottom 30% as EphA2^low^. GSEA^24^ was used to compare the gene set differences between the two groups.

### Bimolecular fluorescence complementation (BiFC) assay

The BiFC assay is based on the reconstitution of a fluorescent protein upon the reassociation of two split nonfluorescent fragments via their linkage to independent interacting proteins. When Venus green fluorescent protein (Venus, enhanced GFP) was cut into two fragments containing either the N-terminal (VN) or C-terminal (VC), neither of the fragments displayed fluorescent property when expressed alone. Coexpression of the two fragments linked to interacting proteins allowed the partial reformation of Venus with the concomitant appearance of the fluorescent signal. Details of the procedure were as described^25^. Briefly, HEK293 cells were transiently co-transfected with VN- and VC-tagged plasmids at a ratio of 1:1 for 48 h. Images were taken with a fluorescence microscope using excitation (480 ± 30 nm) and emission (535 ± 25 nm) filters under the same conditions.

### Cell surface-binding assay

Flag-Wnt1 was transfected into HEK293 cells for 48 h, and then the cell culture medium (Wnt1 CM) was collected and concentrated with Amicon-Ultra-15 filters (Millipore). EphA2, Frizzled2, or an empty vector were transfected into HEK293 cells for 36 h and the cell surface-binding assay was performed as described^26^.

### Proximity ligation assay (PLA)

PLA was carried out using Duolink in situ fluorescence kit (Sigma-Aldrich) according to the manufacturer’s protocol. In brief, HEK293 cells were transfected with pcDNA3.1-EphA2. Forty-eight hours later, cells were incubated with Flag-Wnt1 conditional medium (CM) for an additional 3 h, the cells were fixed with 4% paraformaldehyde and permeabilized with 0.25% Triton X-100, followed by blocking for blockage. Anti-Flag (mouse) and anti-EphA2 (Rabbit) antibodies were added and incubated at 4℃ overnight. Secondary antibodies conjugated with oligonucleotides (Rabbit antibody with PLA probe plus and Mouse antibody with PLA probe minus) were incubated for 1 h at 37 °C after primary antibody. After wash, ligation was taken place for 30 min at 37 °C, followed by amplification with polymerase for 100 min at 37 °C.

### Xenograft, PDX, and drug studies in vivo

All animal care and euthanasia protocols were approved by the Institutional Animal Care and Use Committee of Central South University (Changsha, China). For the cancer cell xenograft study, 4-week-old nude male mice were injected subcutaneously in the hind flanks with 5 × 10^6^ EphA2 shRNA knockdown (or negative control shRNA) AGS cells in 100 μl RPMI-1640 that was mixed with Matrigel (1:1). Once tumors reached 100 mm^3^, mice received 20 mg/kg ALW in 10% 1-methyl-2-pyrrolidinone and 90% polyethylene glycol 300 or the vehicle alone. ALW is a small-molecule tyrosine kinase inhibitor of EphA2 that effectively inhibits EphA2 function in lung cancer and breast cancer models^27–29^. Mice were treated once daily via intraperitoneal injection, and tumors were measured daily with digital calipers. Tumor volume was calculated using the following formula: volume = length × width^2^/2. Each experimental group had five mice.

For PDX (patient derived xenograft) mouse model, ~2 mm^3^ portions of freshly resected gastric tumor tissues were implanted subcutaneously in 4-week-old athymic recipient male NOD/SCID mice. GC001, GC002, GC003, and GC004 gastric cancer-derived lines were established, and the information for the four gastric cancer patients is listed in Supplementary Table S3. Written informed consent was obtained from all patients, and the procedure was approved by the Ethics committee of the Xiangya Hospital, Central South University. When the initial tumors reached 1000–1500 mm^3^ in the mice, tumors were collected, cut into 2-mm^3^ portions, and serially transplanted subcutaneously in 4-week-old recipient male NOD/SCID mice to establish cohorts^29^. Tumors were allowed to grow to ~50–100 mm^3^. The mice were randomly divided into two groups: the control group was injected with PBS, and the ALW group was injected intraperitoneally once daily for 3 weeks with 20 mg/kg ALW in 10% 1-methyl-2-pyrrolidinone and 90% polyethylene glycol 300. Each group had five mice. Tumors were measured four times weekly, and tumor volume was calculated as described above. At the end of the treatment period, tumors were collected and analyzed for proliferation, apoptosis, and microvascular density as described above. All animal procedures and were performed in accordance with institutional guidelines.

### Statistical analysis

Statistical significance was calculated using Prism (GraphPad Software) and SPSS17. All experiments were performed in triplicate. Data represent the mean ± s.d. Statistical differences were assessed with the unpaired Student *t*-test, and *P*-values < 0.05 were considered to reflect statistical significance.

## Results

### EphA2 Enhances Wnt/β-catenin Signaling

To further characterize the effect of EphA2 on Wnt/β-catenin signaling, we employed a well-established Wnt-responsive Top/Fop-flash luciferase reporter assay. We found that EphA2 enhanced Wnt-driven luciferase activity. Conversely, knockdown of EphA2 inhibited Wnt/β-catenin signaling (Fig. 1a). We also observed similar changes in post-transfection expression of *EphA2* and *CTNNB1* (encoding β-catenin) with the *EphA2*-expressing vector in gastric cancer cells BGC823 and AGS (Fig. 1b), which indicated β-catenin is closely regulated by EphA2.

**Fig. 2:**
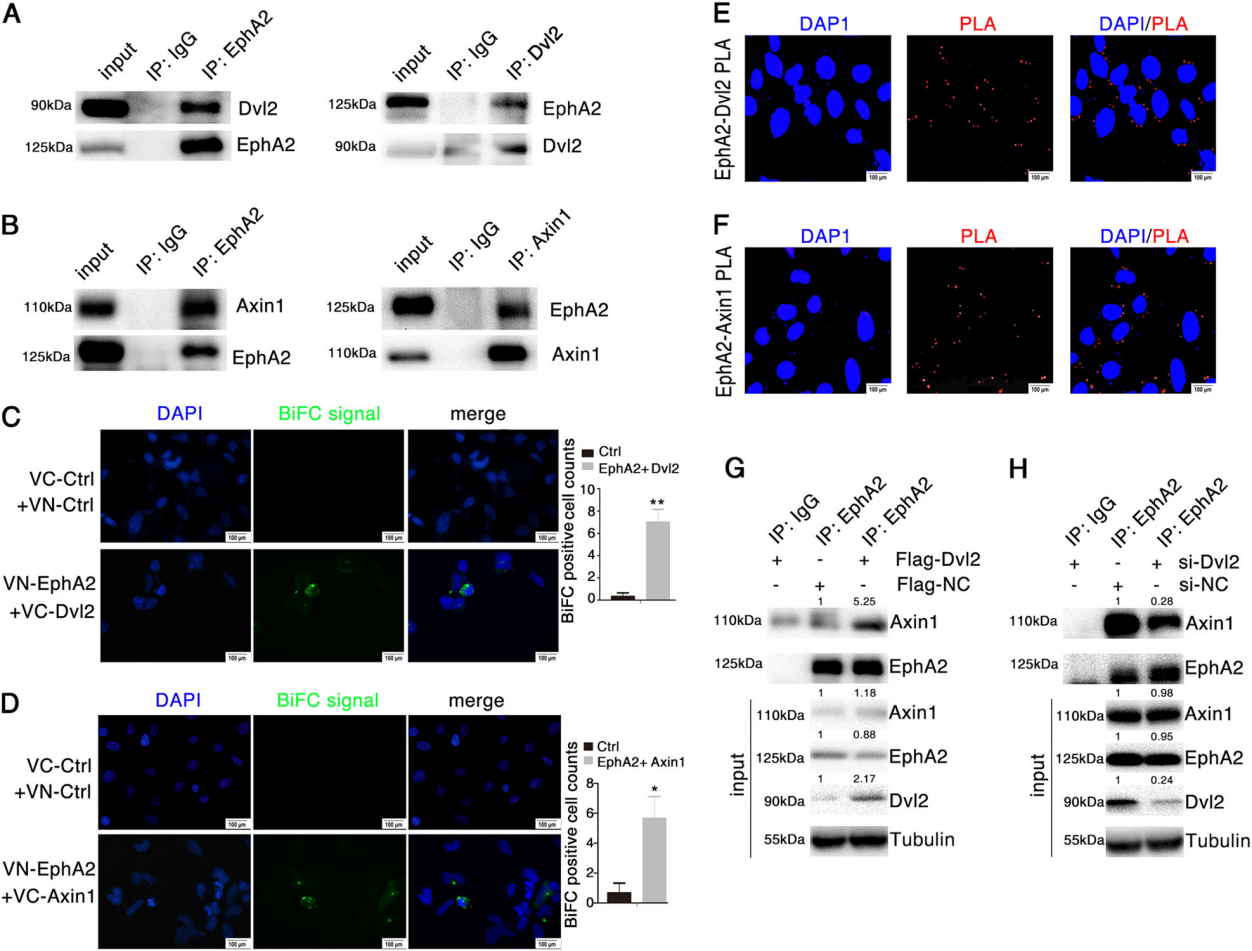
EphA2 interacts with Dvl2/Axin1. **a**, **b** Interaction between endogenous EphA2 with Dvl2 or Axin1 in AGS cells. Western blotting of cell lysates subjected to co-IP with indicated antibodies against EphA2, Dvl2 or Axin1. **c**, **d** BiFC signal analysis by fluorescence microscopy after transient co-expression of **c** VN-EphA2 and VC-Dvl2 and **d** VN-EphA2 and VC-Axin1 in HEK293 cells. **e**, **f** Proximity ligation assays (PLA) for EphA2 and Dvl2/Axin1. Interaction between endogenous EphA2 with Dvl2 (**e**) or Axin1 (**f**) in AGS cells. **g**, **h** Interaction between endogenous EphA2 and Axin1 in AGS cells transfected with Flag-Dvl2 expression vector (**g**) and si-Dvl2 (**h**) and analyzed by co-IP using anti-EphA2. Relative accumulations of proteins in different groups compared with the negative control group are indicated. Significant differences were determined with the Student’s *t*-test. *P < 0.05, **P < 0.01 compared with control group

Through analyzing the Oncomine (http://www.omcomine.org) data^30–32^, we noted significantly elevated *EphA2* expression in gastric cancer tissues compared with corresponding normal tissues, which mirrored the expression profiles of *c-MYC* and *β-catenin* (Supplementary Fig. S1). We extended this finding by analyzing gene expression data for gastric cancer specimens from The Cancer Genome Atlas (TCGA) database^23^, which revealed very similar expression patterns of *EphA2*, *c-MYC*, *CCND1* (encoding cyclin D1), and *CTNNB1* in 295 gastric cancer specimens^23^ (Supplementary Fig. S2A). We also found obvious positive correlations between the expression of *EphA2* and each of *c-MYC*, *CCND1*, *CTNNB1* (Supplementary Fig. S2B). Gene set enrichment analysis (GSEA) revealed gene sets such as “MYC-Targets” is strongly enriched in EphA2^high^ gastric cancer specimens compared with EphA2^low^ specimens (see Materials and Methods; Supplementary Fig. S2C). *c-MYC* and *CCND1* are downstream target genes of Wnt signaling, which suggests that EphA2 overexpression indeed enhances Wnt signaling in gastric cancer.

### EphA2 binds to Wnt1

To clarify the exact mechanisms of the EphA2 regulation of Wnt/β-catenin signaling in gastric cancer, we analyzed the structure of EphA2, and found that EphA2 shares a cysteine-rich domain (CRD) with Frizzled, a receptor for Wnt ligands. We thus performed a cell surface-binding assay^26^ to explore whether EphA2 also interacts with Wnt1. Cells were transfected with either pcDNA3.1-EphA2, or Frizzled2 (a well known Wnt receptors, as a positive control here). After incubation with Flag-Wnt1 conditional medium (CM), Wnt1 bound to cells transfected with EphA2 or Frizzled2, whereas no or little Wnt1 binding was detected for empty vector-transfected cells, or soluble Ephrin A1 blocked cells (Fig. 1c). Moreover, proximity ligation assay (PLA) further confirmed the interaction between EphA2 and Wnt1, and the interaction was inhibited by EphrinA1, an EphA2 ligand that can block EphA2’s downstream oncogenesis function (Fig. 1d). To further validate the interaction between Wnt1 and EphA2, pcDNA3.1-EphA2 and Flag-Wnt1 were co-expressed in HEK293 cells, and the interaction between EphA2 and Wnt1 was verified by co-immunoprecipitation (co-IP) (Fig. 1e). The co-localization of EphA2 and Wnt1 at the cell surface was also demonstrated by immunostaining (Supplementary Fig. S3A). To test for direct binding between Wnt1 and EphA2, we performed a GST pull-down assay by incubating purified GST-Wnt1 with EphA2. After pull-down with Glutathione-beads, EphA2 was detected specifically in the GST-Wnt1–bound beads (Fig. 1f), which indicated that EphA2 was bound directly by Wnt1. To clarify whether EphA2 may work through cooperation with Frizzled and LRP6 instead of directly bind to Wnt1, we performed co-IP assay which showed that EphA2 can not bind to Frizzled2 and LRP6 (Supplementary Fig. S3B), and immunostaining assay which showed that EphA2 was not co-localized with Frizzled2 and LRP6 at the cell surface (Supplementary Fig. S3C, D). Taken together, these results suggested that EphA2 functions as a Wnt1 receptor.

### EphA2 interacts with Dvl2/Axin1

The best-characterized branch of the Wnt signaling pathway is mediated by the canonical Wnt/Frizzled-LRP6/Dvl2/Axin1-GSK3β-β-catenin. Dvl2 is the scaffold protein that relays Wnt signaling by bridging receptor and adapter proteins in both the canonical and non-canonical Wnt pathways^33^. Axin1 acts as a scaffold protein for the β-catenin destruction complex and is an important negative regulator of Wnt/β-catenin signaling^34^. As EphA2 promotes Wnt/β-catenin signaling, we hypothesized that EphA2 may relay Wnt signaling through Dvl2 and Axin1. As shown in Fig. 2a, b, endogenous EphA2 interacted with Dvl2 and Axin1 in AGS cells, as evidenced by reciprocal co-IP assays. Similarly, exogenous EphA2 also interacted with Dvl2 (Supplementary Fig. S4A, B), and Axin1 (Supplementary Fig. S4C, D).

**Fig. 3:**
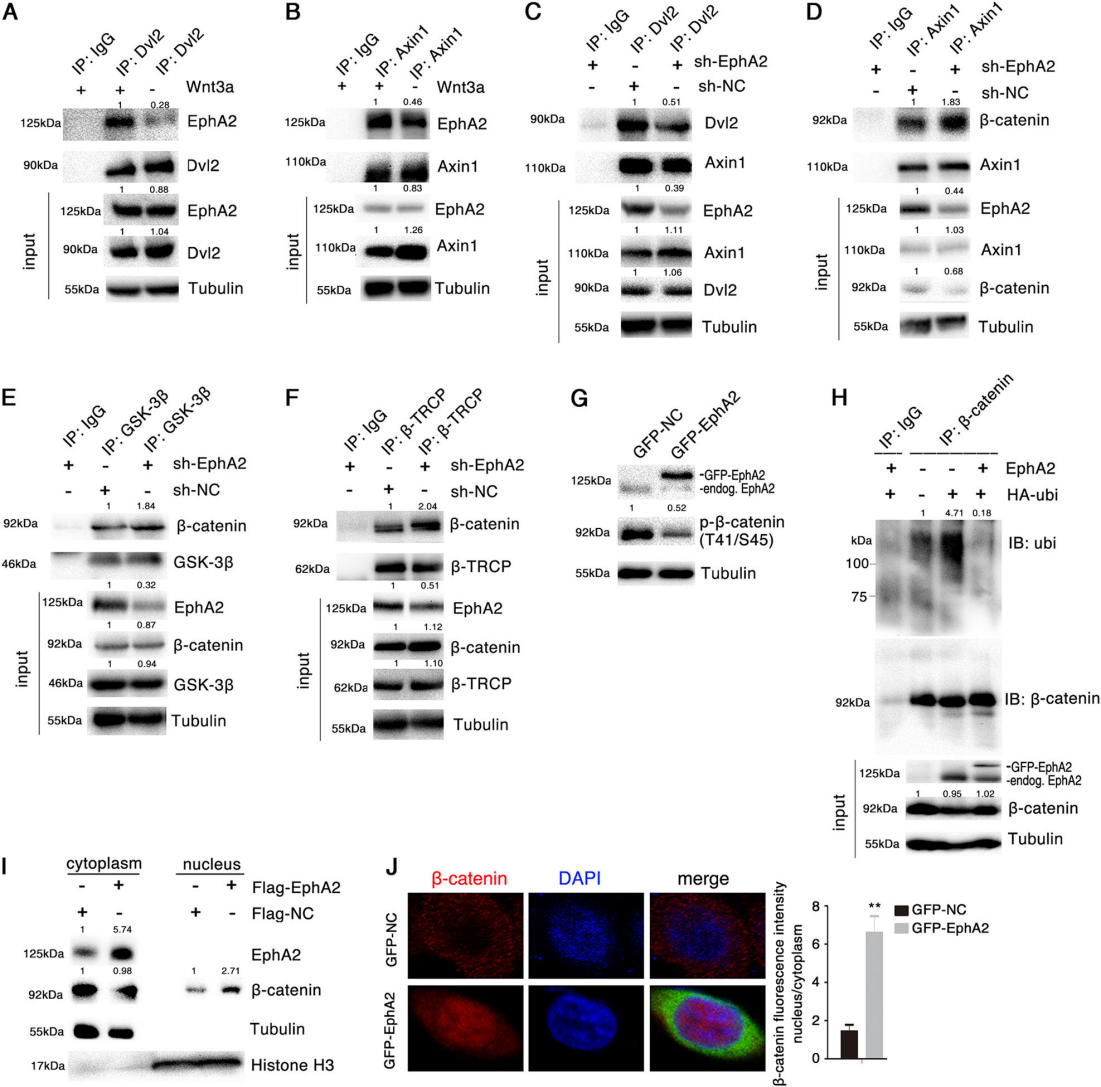
EphA2 destabilizes the β-catenin destruction complex and promotes its nuclear accumulation. **a**, **b** Interaction between endogenous EphA2 and Dvl2 (**a**) or Axin1 (**b**) with or without Wnt3a stimulation in AGS cells, assayed by co-IP. **c**–**f** Interaction between endogenous EphA2 and β-catenin destruction complex. A stable AGS cell line in which EphA2 expression was inhibited via EphA2-specific shRNA virus, and cell lysates were subjected to co-IP followed by Western blotting for indicated antibodies. **g** Phosphorylation of β-catenin at residues Thr41/Ser45 after transfection of AGS cells with GFP-EphA2 for 48 h. **h** Levels of β-catenin ubiquitination in AGS cells after transfection with the indicated expression vectors for 48 h followed by treatment with the proteasome inhibitor MG132 for 4 h before harvesting. β-catenin was immunoprecipitated with anti-β-catenin and subjected to Western blotting with anti-ubiquitin and anti-β-catenin. **i** Relative levels of β-catenin and EphA2 at 48 h post-transfection with EphA2 or negative control expression vectors in AGS cells assessed with Western blotting of nuclear and cytoplasmic proteins. **j** Effect of EphA2 overexpression on the subcellular localization of β-catenin in AGS cells monitored by immunofluorescence. Relative accumulations of proteins in different groups compared with the negative control group are indicated. Significant differences were determined with the Student’s *t*-test. **P < 0.01 compared with control group

To further verify the interaction between EphA2 and Dvl2 in Wnt signaling, we established a bimolecular fluorescence complementation (BiFC) system for visualizing and analyzing the interaction between EphA2 and Dvl2. Co-expression of VN-tagged EphA2 and VC-tagged Dvl2 (two fragments containing either the GFP N-terminal, VN or GFP C-terminal, VC) in cells resulted in a significantly more intense BiFC signal compared with empty-vector control (Fig. 2c). Co-expression of EphA2 and Axin1 yielded a similar BiFC result (Fig. 2d). Moreover, proximity ligation assay (PLA) also revealed the endogenous interaction existed between EphA2 and Dvl2/Axin1 in AGS cells (Fig. 2e, f). In parallel, immunofluorescence results also suggested that EphA2 co-localized with Dvl2 and Axin1 (Supplementary Fig. S3E, F) at the cell surface.

To determine the region of EphA2 protein involved in binding to Dvl2 and Axin1, we generated two series of *EphA2* mutant fusion proteins tagged at their N-terminus with Flag or GST (Supplementary Fig. S4E). The GST pull-down assay indicated that the tyrosine kinase domain of EphA2 was critical for binding Dvl2 but not Axin1 (Supplementary Fig. S4G). co-IP confirmed that the Flag-EphA2 expression vector lacking the tyrosine kinase domain could not bind Dvl2 (Supplementary Fig. S4F). Moreover, the qPCR experiment further showed that the EphA2 with deletion of tyrosine kinase domain significantly reduced the expression of *c-MYC* and *CCND1* (Supplementary Fig. S4H). Collectively, these results suggested that the tyrosine kinase domain of EphA2 interacts with Dvl2 but not Axin1. Therefore, we speculated that Dvl2 may mediate the interaction between EphA2 and Axin1. Co-IP assays revealed that, as expected, Dvl2 overexpression markedly enhanced the EphA2-Axin1 interaction in AGS and BGC823 cells (Fig. 2g, Supplementary Fig. S4I), whereas silencing of Dvl2 had the opposite effect (Fig. 2h, Supplementary Fig. S4J), which suggested that the proteins form an EphA2/Dvl2/Axin1 complex with Dvl2 at the center. To rule out that EphA2 interacts only physically, i.e., not functionally, with Dvl2, we performed co-IP assays to verify that EphA2 is indeed a receptor for Wnt/β-catenin signaling. Stimulation of AGS and BGC823 cells with Wnt3a significantly enhanced the interaction between EphA2/Dvl2 and EphA2/Axin1 (Fig. 3a, b, Supplementary Fig. S5A, B), suggesting that EphA2 indeed acts as a functional receptor for the Wnt/β-catenin pathway.

**Fig. 4:**
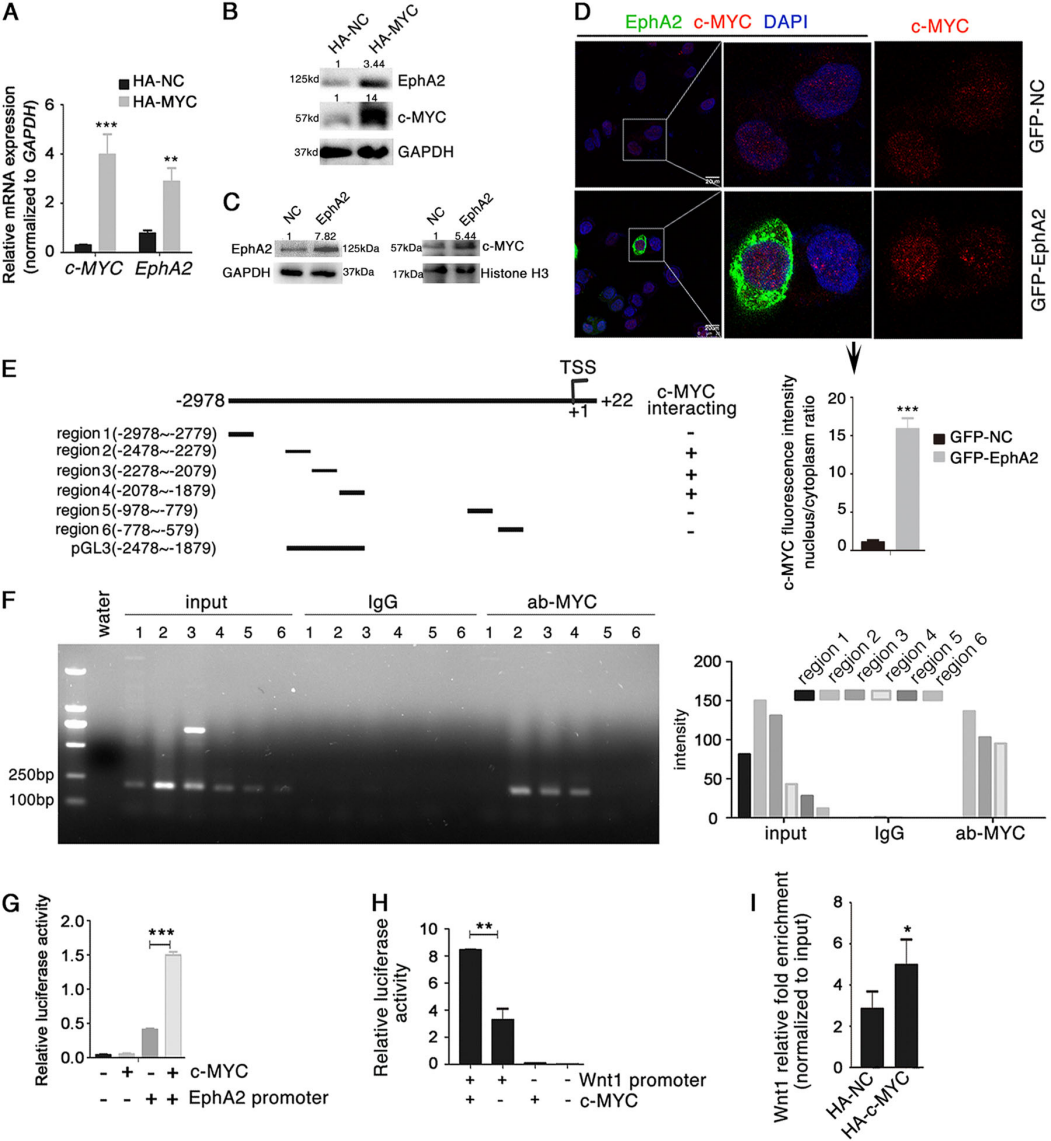
*EphA2* is a c-MYC target gene. **a**, **b** c-MYC and EphA2 mRNA expression (**a**) and protein levels (**b**) in AGS cell lysates after transfection with the HA-tagged c-MYC expression vector. **c** Expression of nuclear and cytoplasmic c-MYC at 48 h post-transfection with Flag-EphA2 expression vector in AGS cells assayed by Western blotting. **d** Effect of EphA2 overexpression on the subcellular localization of c-MYC in AGS cells monitored by immunofluorescence. Cells were transfected with GFP-EphA2 or GFP-NC vector. **e** Schematic diagram of the truncated forms of the putative EphA2 promoter region and their interaction with c-MYC. **f** Identification of c-MYC-binding regions in the *EphA2* promoter in AGS cells by ChIP using anti-c-MYC or normal rabbit IgG (negative control) (left panel). Quantification of band intensities of the PCR products is in right panel. **g** EphA2 transcription activity assayed in HEK293 cells transfected with the c-MYC expression vector and pGL3-EphA2-promotor luciferase reporter plasmid. **h** Wnt1 promoter-driven luciferase activity analyzed after co-transfection of HEK293 cells with the c-MYC expression vector and pGL3-Wnt1-promotor vector. **i** Identification of c-MYC-binding sites in the Wnt1 promoter in AGS cells by ChIP using anti-c-MYC. Normal rabbit IgG was used as a negative control. Relative accumulations of proteins in different groups compared with the negative control group are indicated. Significant differences were determined with the Student’s *t*-test. *P < 0.05, **P < 0.01, ***P < 0.001 compared with control group

### EphA2 destabilizes the β-catenin destruction complex and promotes its nuclear accumulation

Dvl2 and Axin1 serve as scaffold for the β-catenin destruction complex bringing together APC, β-catenin, CK1, GSK3β, and the ubiquitin E3 ligase β-TRCP. In this context, we speculated that EphA2 destabilizes the β-catenin destruction complex by recruiting Axin1 to the membrane and by binding to Dvl2. To test this possibility, we first performed co-IP. As expected, EphA2 overexpression markedly enhanced the endogenous Dvl2-Axin1 interaction in AGS cells (Supplementary Fig. S5E). In parallel, we found that EphA2 significantly inhibited β-catenin interaction with Axin1, GSK3β, and β-TRCP (Supplementary Fig. S5F–H). To confirm that EphA2 disrupts the interaction between the components of the destruction complex, we established a stable AGS cell line in which EphA2 function was inhibited via expression of an EphA2-specific shRNA. We confirmed that the endogenous interaction between Dvl2 and Axin1 was inhibited upon EphA2 downregulation, and β-catenin-Axin1, β-catenin-GSK3β, and β-catenin-β-TRCP interactions were indeed enhanced upon EphA2 downregulation (Fig. 3c–f).

This finding raised a possibility that EphA2 can stabilize β-catenin. We thus examined the effect of EphA2 on the level of phosphorylated β-catenin, which is critical to its recognition by the ubiquitin E3 ligase β-TRCP and subsequent degradation by the proteasome. Overexpression of EphA2 decreased the phosphorylation of β-catenin at Thr41/Ser45 sites that are phosphorylated by CK1α and GSK3β (Fig. 3g, Supplementary Fig. S5C); and inhibited β-catenin ubiquitination (Fig. 3h), suggesting that EphA2 promotes the stabilization of β-catenin. EphA2 overexpression increased the nuclear localization of β-catenin, whereas there was little or no effect on the cytoplasmic level of β-catenin (Fig. 3i, Supplementary Fig. S5D). β-catenin localized mainly in the cytoplasm in normal control cells, but the protein underwent substantial translocation to the nucleus upon EphA2 overexpression in AGS cells (Fig. 3j. Supplementary Fig. S5I). These results suggested that EphA2 destabilizes the β-catenin destruction complex by promoting the interaction between Dvl2 and Axin1; and reduces the phosphorylation of β-catenin, thereby preventing its ubiquitination and promoting β-catenin nuclear accumulation.

### EphA2 governs a feed-forward loop in Wnt signaling

We found that, interestingly, information from several databases (GeneCards, Jasper) showed that the transcription factor c-MYC (also a target gene of Wnt signaling) binds to the promoter of *EphA2*, which suggested a possibility that once activated, EphA2 may orchestrate a feed-forward loop to propagate Wnt signaling. Indeed, overexpression of c-MYC markedly increased the protein and mRNA levels of EphA2 (Fig. 4a, b); and EphA2 promoted c-MYC translocation to the nucleus (Fig. 4c, d).

**Fig. 5:**
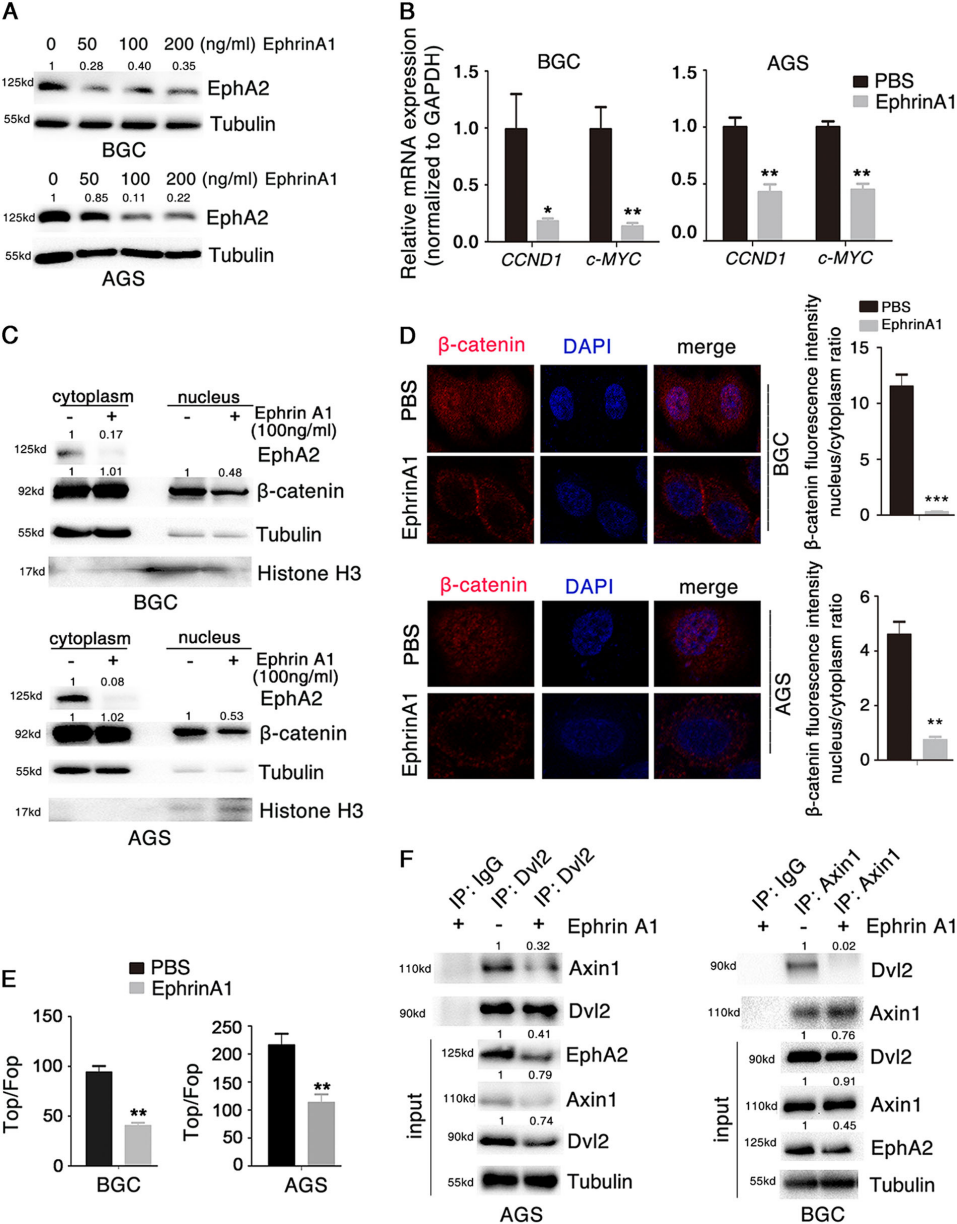
EphrinA1 inhibits Wnt/β-catenin signaling. **a** EphA2 levels assayed by Western blotting of lysates of BGC823 and AGS cells stimulated with EphrinA1 at different concentrations for 4 h. **b** *CCND1*, and *c-MYC* expression as assessed with real-time qPCR after stimulation of cells with EphrinA1 (100 ng/ml) for 4 h. **c** Relative levels of nuclear and cytoplasmic β-catenin and EphA2 assessed with Western blotting at 4 h post-stimulation with EphrinA1 (100 ng/ml). **d** Effect of EphrinA1 (100 ng/ml, 4 h) on the subcellular localization of β-catenin monitored by immunofluorescence. **e** Activity changes in Wnt/β-catenin signaling upon stimulation with EphrinA1 (100 ng/ml, 4 h) as assessed with the TOP-flash/FOP-flash luciferase reporter. **f** Interaction between endogenous Dvl2 and Axin1 in the presence of EphrinA1 in AGS (left) or BGC823 (right) cells, analyzed by co-IP. Relative accumulations of proteins in different groups compared with the negative control group are indicated. Significant differences were determined with the Student’s *t*-test. *P < 0.05, **P < 0.01, ***P < 0.001 compared with control group

We next identified a 3000-bp region spanning positions –2978 to +22 relative to the transcription start site as the putative promoter of *EphA2* through UCSC (http://genome.ucsc.edu/). We generated a series of primers corresponding to the putative *EphA2* promoter (Fig. 4e), and ChIP experiment demonstrated that c-MYC bound to regions 2, 3, and 4 of the *EphA2* promoter (Fig. 4f). To validate the interaction between c-MYC and the promoters of *EphA2*, we inserted the putative c-Myc-binding region (regions 2–4) of the *EphA2* promoter in the pGL3-enhancer vector (Fig. 4e), a luciferase-based promoterless plasmid. The luciferase activity increased significantly upon co-transfection with the c-MYC-expressing vector as compared with mock transfections (Fig. 4g). We also found that c-MYC bound to the putative *Wnt1* promoter based on luciferase reporter activity and ChIP data (Fig. 4h, i). These results suggested that Wnt ligands interact with EphA2 and thus activate Wnt/β-catenin signaling, inducing transcription of downstream target genes such as *c-MYC*. c-Myc itself functions as a transcription factor to bind to the promoters of *EphA2* and *Wnt1* and initiate their transcription, which forms a feed-forward loop for the continuous activation of Wnt signaling.

### EphrinA1 inhibits Wnt/β-catenin signaling

Data presented thus far provided strong evidence that EphA2 functions in promoting Wnt signaling in gastric cancer cells. We therefore changed our perspective and asked whether the EphA2 ligand EphrinA1 also affects Wnt/β-catenin signaling. First, we found that incubation of cells with different concentrations of EphrinA1 decreased the levels of EphA2 (Fig. 5a), and *CCND1* and *c-MYC* (Fig. 5b), consistent with previous reports showing an inverse correlation between EphA2 level and EphrinA1 in breast cancer cells^35^. Next, EphrinA1 induced a significant decrease in β-catenin level in the nuclear, but not cytoplasmic fraction (Fig. 5c). Third, in EphrinA1-treated cells, the intensity of β-catenin positively stained cells decreased compared with control cells, and the β-catenin proteins were located mainly in the cytoplasmic fraction (Fig. 5d). Fourth, cells treated with EphrinA1 exhibited a significant inhibition of Top luciferase activity, which indicated reduced transcriptional activity of Wnt signaling (Fig. 5e). Moreover, treatment of cells with EphrinA1 inhibited the interaction between Dvl2 and Axin1 (Fig. 5f), implying enhanced activity for the β-catenin destruction complex. These results revealed a suppressive role for EphrinA1 in Wnt/β-catenin signaling.

**Fig. 6:**
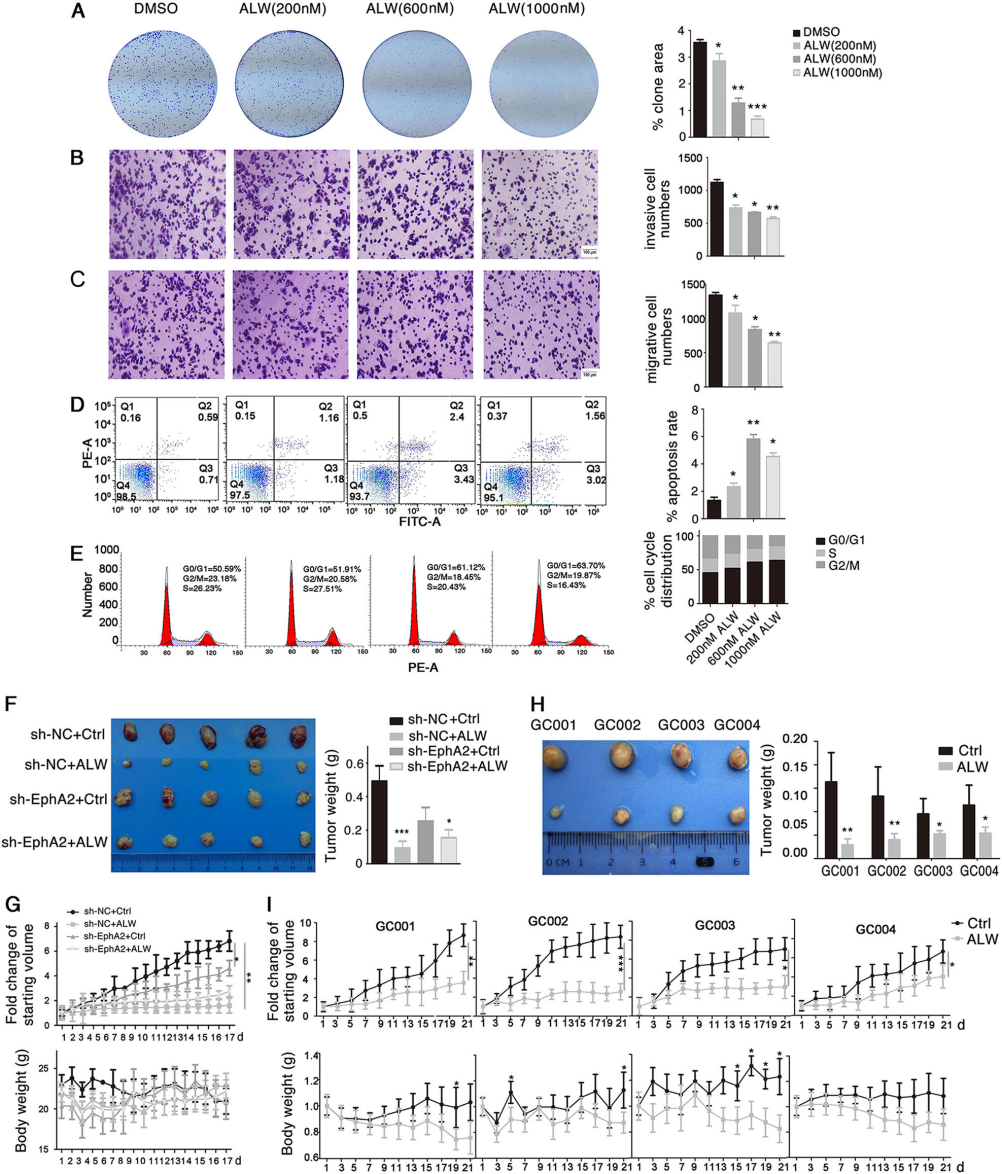
ALW inhibits gastric cancer tumor growth by targeting EphA2. **a–e** ALW suppresses gastric tumorigenesis in AGS cells in vitro in a dose-dependent manner. Cell parameters were determined with **a** cell clone-formation assay, **b**, **c** transwell Matrigel assays for **b** cell invasion and **c** migration, **d** apoptosis analysis, and **e** cell-cycle analysis. Quantifications are shown to the right in each panel. **f**, **g** sh-NC or sh-EphA2 AGS cells (5 × 10^6^) were injected subcutaneously into the dorsal flanks of nude mice. Tumors were allowed to grow to ~100 mm^3^ before administration of 20 mg/kg ALW (or control, vehicle alone) via intraperitoneal injection once daily. **f** Comparison of tumor graft size and weight in nude mice. **g** Tumor volume (upper panel) and body weight (*lower panel*) were measured daily. **h**, **i** Four independent gastric cancer PDX lines were implanted into NOD/SCID mice. When tumors reached ~50–100 mm^3^, tumor-bearing mice were randomly treated with vehicle control or ALW for 3 weeks. **h** Comparison of PDX tumor graft size and weight in NOD/SCID mice. **i** Tumor volume (upper panel) and body weight (lower panel) were measured. Significant differences were determined by the Student’s *t*-test. *P < 0.05, **P < 0.01, ***P < 0.001, compared with control group

### Targeting EphA2 impairs tumor growth in vitro and in clinically relevant gastric cancer models in vivo

Considering the significant roles of EphA2 and Wnt signaling in tumor development, we determined the impact of targeting EphA2 on gastric cancer by using a specific EphA2 inhibitor ALW-II-41-27^27–29^. ALW caused a decrease in EphA2 level in a dose-dependent manner (Supplementary Fig. S6A). CCK8 assays over a 72-h time course revealed that ALW significantly impaired the proliferation of AGS and BGC823 cells (Supplementary Fig. S6B). We performed assays for tumor cell clone formation, cell cycle, apoptosis, migration and invasion, and found that ALW decreased the proliferation and invasiveness of both AGS (Fig. 6a–e) and BGC823 cells (Supplementary Fig. S7A–E).

**Fig. 7:**
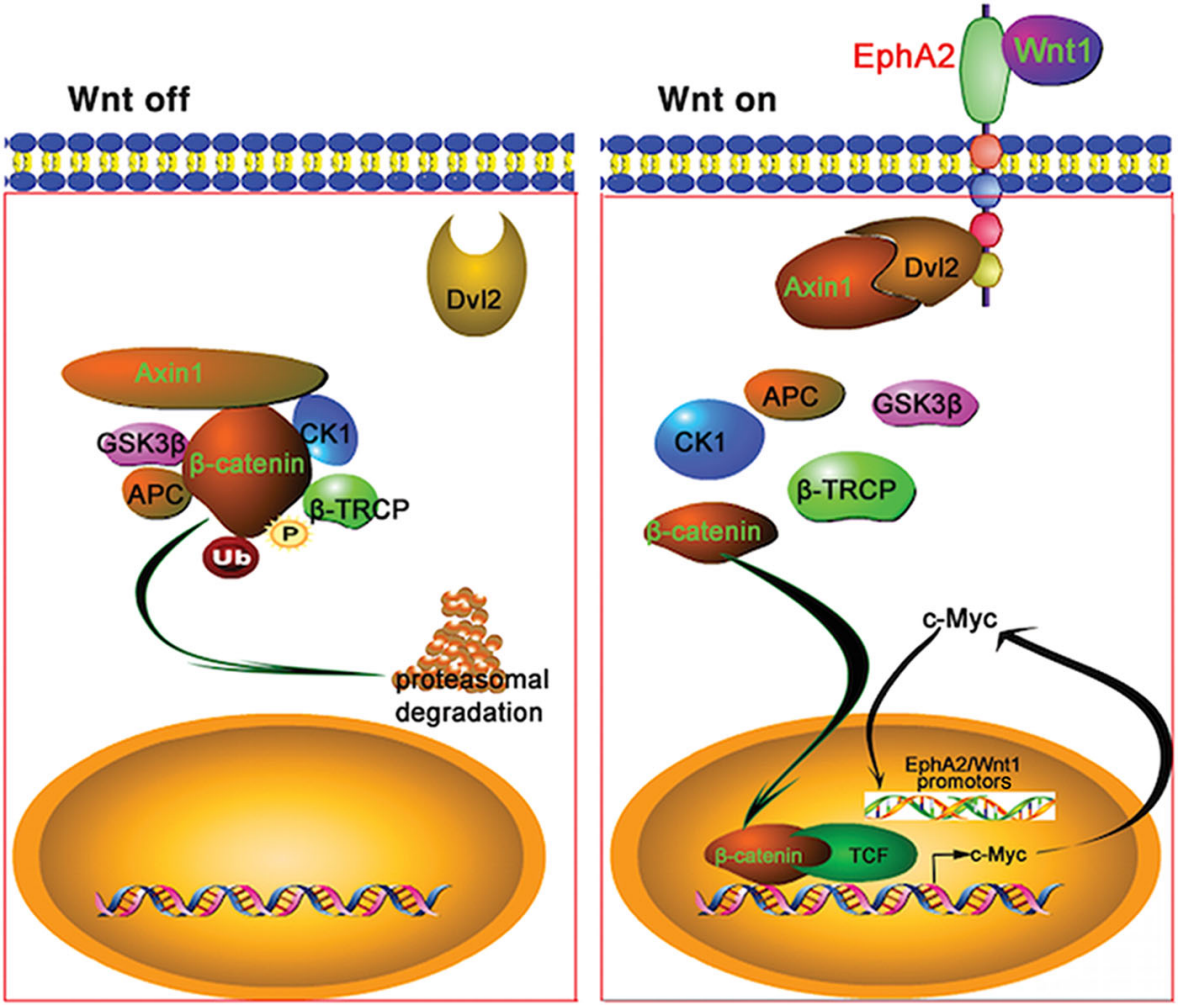
Model depicting the main molecular mechanisms that function in EphA2-mediated Wnt signaling

We then investigated the effect of ALW on Wnt signaling using the Top/Fop-flash luciferase reporter assay and found that treatment with ALW largely decreased the Wnt-driven luciferase activity (Supplementary Fig. S7C) as well as the expression of *CCND1* and *c-MYC*, target genes of Wnt signaling (Supplementary Fig. S6D). Moreover, we further found that ALW treatment also blocked the interaction of EphA2 with Dvl2/Axin1 and reduce potential EphA2-EphrinA1 interaction (Supplementary Fig. S6E--F).

We established stable AGS and BGC823 cell lines in which the endogenous EphA2 expression was silenced by an shRNA-virus targeting EphA2. We treated the sh-NC (i.e. negative control shRNA) and sh-EphA2 cells with ALW or DMSO (as negative control) and evaluated whether the inhibitory effect of ALW on gastric cancer cells was dependent on the EphA2 level. Compared with DMSO treatment, ALW significantly reduced the proliferation of both AGS and BGC823 cells, but the decrease was less prominent in the sh-EphA2 group than the sh-NC group of cells (Supplementary Fig. S8). These results implied that EphA2 is a specific target of ALW, which is consistent with EphA2 being a functionally important target of ALW in non-small-cell lung cancer^27^.

We next investigated the role of ALW in gastric cancer development using a xenograft nude mouse model. The nude mice were randomly divided into four groups: sh-NC + Ctrl, sh-NC + ALW, sh-EphA2 + Ctrl, and sh-EphA2 + ALW. We treated 100 mm^3^ sh-NC and sh-EphA2 AGS xenograft tumors with ALW (or vehicle alone as negative control) via intraperitoneal injection. Compared with vehicle treatment, administration of ALW to tumor-bearing animals for 17 days significantly inhibited tumor growth in vivo, but the decrease was less prominent in the sh-EphA2 group than in the sh-NC group (Fig. 6f, g top panel). Body weight remained stable during the course of the study (Fig. 6g lower panel). Staining of the tumors with hematoxylin and eosin revealed no significant differences among the four groups with respect to tumor pathology (Supplementary Fig. S9A). Analysis of tumor lysates revealed that EphA2 protein levels decreased upon ALW treatment. Moreover, ALW also decreased the expression of *CCND1*, *c-MYC* and β-catenin (Supplementary Fig. S9B, C, F). ALW treatment showed a significant increase in apoptosis as assayed by TUNEL staining especially in sh-NC AGS tumors, whereas this phenomenon was compromised in sh-EphA2 tumors (Supplementary Fig. S9D). ALW treatment also inhibited the cell proliferation and tumor microvessel density (as measured by Ki67 and CD31 staining) in sh-NC AGS tumors, and these inhibition abilities were impaired in sh-EphA2 tumors (Supplementary Fig. S9E).

To investigate the impact of EphA2 targeting on clinically relevant models of gastric cancer, we built a PDX mouse model from tissues from four gastric cancer patients. The PDX tissues were resected from donor animals and transplanted into NOD/SCID mice. When tumors reached ~50–100 mm^3^, tumor-bearing mice were randomly divided into two groups, treated with ALW or vehicle (as control) via intraperitoneal injection. Administration of ALW to tumor-bearing mice for 3 weeks significantly inhibited tumor growth (Fig. 6h, i upper panel), whereas body weight remained stable (Fig. 6i lower panel). These data indicate that pharmacologic targeting of EphA2 may be an effective strategy in gastric cancer therapy.

## Discussion

Although EphA2 has been extensively studied as a regulator of many signaling pathways^1^, in most circumstances EphA2 works as a kinase to influence downstream signaling. In this study, we identified EphA2 as a receptor for Wnt ligands, and this interaction suggests that EphA2 is indeed a regulator of Wnt/β-catenin signaling. LDL receptor–related protein (LRP) and Frizzled are canonical co-receptors in canonical Wnt signaling^36^. Frizzled binds Wnt proteins through its CRD^37^, and EphA2 also has this domain. We first guessed whether EphA2 binds to Wnt1 by forming a co-receptor with Frizzled/LRP6. However, our data show that there is no interaction between EphA2 and Frizzled2/LRP6 (Supplementary Fig. S3B--D), suggesting that EphA2 binds with Wnt1 is an independent event. In some recent studies, other receptors of Wnts have also been reported. Ye et al. reported that the cell membrane protein CD146 acts as a receptor for Wnt5a to regulate cell motility and convergent extension by relaying Wnt5a signaling to Dvl^38^. Dent et al. revealed that the receptor for activated C kinase 1 (RACK1) inhibits Dvl2/Axin1 recruitment to the membrane, which stabilizes the β-catenin destruction complex by binding Dvl2/Axin1^39^. Dvl2 is the scaffold protein that relays Wnt signaling by bridging receptor and adapter proteins in both the canonical and non-canonical Wnt pathways^40^. In previous studies, certain Dvl proteins interact with EphB2^41^. Our present results reveal that EphA2 conveys Wnt ligand signaling by interacting with Dvl2/Axin1, and this process is similar to the Frizzled-mediated membrane recruitment of Dvl2, which interacts with Axin through its DIX domain to interfere with the function of downstream β-catenin^15,42^. Our results indicate that EphA2 plays a similar role to the Frizzled receptor in regulating Wnt/β-catenin signaling. However, when, where, and how Wnt ligands use different receptors to relay signaling to the downstream effectors—and what changes take place when EphA2 binds to Wnt ligands—are issues that will necessitate further investigation.

The transcription factor c-MYC is a vital downstream target gene of the Wnt/β-catenin pathway, and c-MYC mediates transcription of many oncogenes that regulate cell proliferation, invasion, and metabolism^43^. Loss or inhibition of EphA2 results in reduced expression of *c-MYC*; conversely, EphA2 gain-of-function had the reverse effect. *c-MYC* overexpression rescued proliferation defects induced by the loss of EphA2^29^. In this study, we demonstrated that EphA2 greatly increased the expression of c-MYC and that c-MYC significantly enhanced the transcription of *EphA2* by directly binding to the *EphA2* promoter. We identified that c-MYC directly binds to the *Wnt1* promoter. These new data suggest that EphA2 may function to coordinate a feed-forward loop that ensures EphA2 effectiveness in regulating Wnt/β-catenin signaling (Fig. 7). These observations are similar to previous reports that proposed a conditional feedback loop that regulates the Ras-Raf-MAPK pathway through EphA2^35,44^.

EphA2 is the primary receptor for EphrinA1. Bidirectional signaling between the Ephrin and Eph proteins is involved in multiple physiological processes^1,45^. Eph forward signaling depends on Ephrin binding, which induces Eph receptor clustering, auto-phosphorylation, endocytosis, and proteolytic cleavage^1,46^. Most reports have demonstrated that EphA2 mediates ligand-dependent inhibition and ligand-independent promotion of tumor migration and invasion^47^. However, one study reported that EphrinA1 promotes the malignant progression of intestinal tumors in APC^Min/+^ mice^48^. These reports underscore the complexity of Ephrin–Eph bidirectional signaling. In this study, we found that stimulation with exogenous EphrinA1 inhibited Wnt/β-catenin signaling and reduced EphA2 level, however, the detailed mechanism of EphrinA1 regulation of Wnt/β-catenin remains to be established.

Previous studies showed that ALW inhibited EphA2 function in lung and breast cancers^27–29^. In this study, we identified ALW, as an EphA2 small-molecule inhibitor, can cause gastric cancer cell regression both in vitro and in vivo. Notably, our data indicated that ALW-induced gastric cancer cell regression was much significant in sh-NC tumors comparing to sh-EphA2 (i.e., EphA2-silenced) tumors, implying that tumor inhibition function of ALW is EphA2-dependent, at least partly.

## Electronic supplementary material

Supplementary Tables and Figures
