# Supplementary material for: EPH receptor A2 governs a feedback loop that activates Wnt/β-catenin signaling in gastric cancer
Source: Cell Death Dis. 2018 Nov 19;9(12):1146. doi: 10.1038/s41419-018-1164-y (PMC6242896; doi:10.1038/s41419-018-1164-y)
Supplement: Supplementary file 1 — Supplementary Tables and Figures [file 41419_2018_1164_MOESM1_ESM.docx]

**Supplementary Information**

**Supplementary Table S1. Primer sequence for real-Time PCR**

| Gene | Primer(Forward) | Primer(Reverse) |
| --- | --- | --- |
| *EphA2* | TGGCTCACACACCCGTATG | GTCGCCAGACATCACGTTG |
| *β-catenin* | CATCTACACAGTTTGATGCTGCT | GCAGTTTTGTCAGTTCAGGGA |
| *CCND1* | GCTGCGAAGTGGAAACCATC | CCTCCTTCTGCACACATTTGAA |
| *LEF* | TGCCAAATATGAATAACGACCCA | GAGAAAAGTGCTCGTCACTGT |
| *c-Myc* | GGCTCCTGGCAAAAGGTCA | CTGCGTAGTTGTGCTGATGT |
| *GAPDH* | GGAGCGAGATCCCTCCAAAAT | GGCTGTTGTCATACTTCTCATGG |

**Supplementary Table S2. Primers for ChIP enrichment detection at selected regions in Figure 4E**

| Name | Sequence | Product Size (bp) |
| --- | --- | --- |
| Rigion1 | F: ccctgccctatgctctcagc | 200 |
|  | R: ttgcgagttctgtgttgcgt |  |
| Rigion 2 | F: caagtgatctatctgcctcg | 200 |
|  | R: catgtgagttgtgtggaagc |  |
| Rigion 3 | F: caaaacacttatccaaacca | 200 |
|  | R:catgtaaaccacaggggcca |  |
| Rigion 4 | F: ctgagatttcatgcgtgctt | 200 |
|  | R: tatttgtgagtgtgccatac |  |
| Rigion 5 | F: tgccacagttgctcttcctg | 200 |
|  | R: aggacacctgcctaccacct |  |
| Rigion 6 | F:ccaaactgtcaaaacctatc | 200 |
|  | R: atccccatgttacagaagag |  |

**Supplementary Table S3. Patient characteristics for PDX models in Figure 6H**

| Sample # | GC001 | GC002 | GC003 | GC004 |
| --- | --- | --- | --- | --- |
| Age | 34 | 63 | 62 | 78 |
| Sex | female | male | female | male |
| TNM classification | T_4_N_3_M_0_ | T_2_N_0_M_0_ | T_4_N_3_M_1_ | T_4_N_3_M_0_ |
| Larren classification | diffuse | mixed (diffuse and intestinal) | diffuse | diffuse |
| Lymphocytes infiltrate | +++ | - | +++ | ++ |
| Pathology | adenocarcinoma | adenocarcinoma | adenocarcinoma | adenocarcinoma |

Gastric cancer samples were classified under the Lauren’s criteria; lymphocytic infiltration is indicated as +mild, ++ moderate, and +++ severe.

**Supplementary Figures**


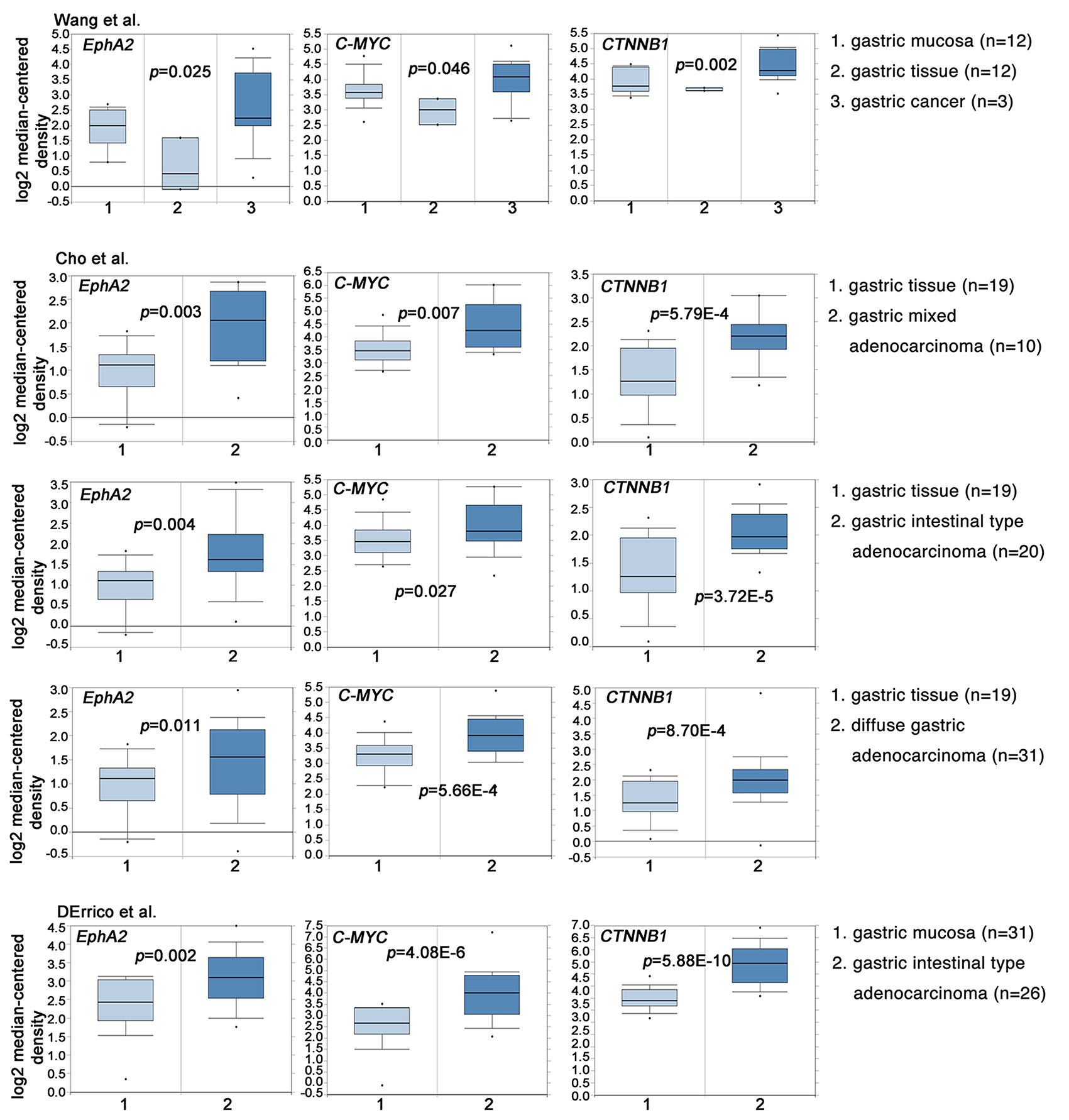


**Figure S1.** Relative mRNA levels of *EphA2*, *c-MYC*, and *CTNNB1* (encodes β-catenin) analyzed in normal gastric tissues and gastric carcinoma tissues using different Oncomine databases (https://www.oncomine.org/resource/login.html)^1-3^.


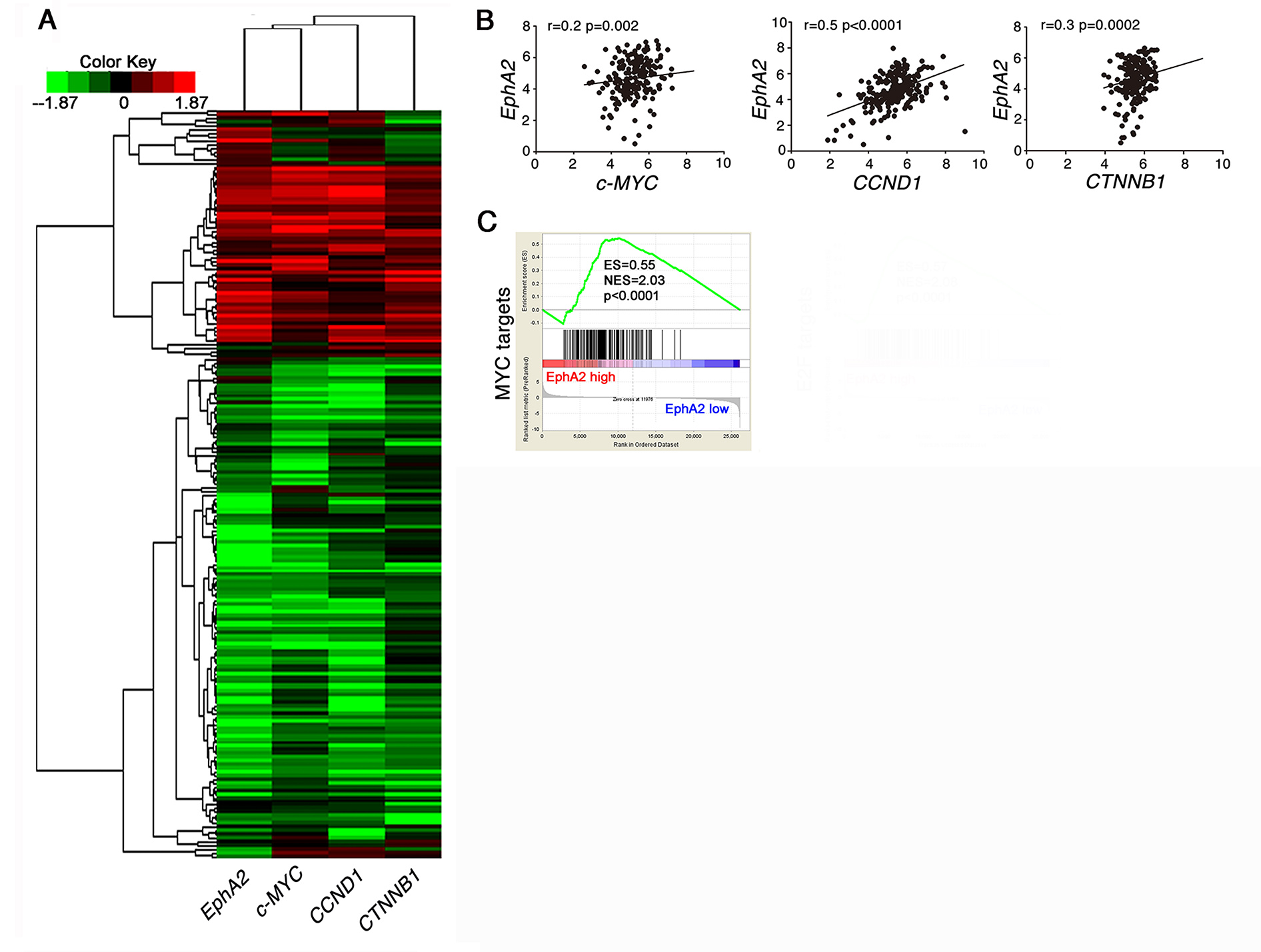


**Figure S2.** **(A)**.Heatmap analysis of the expression patterns of *EphA2*, *c-MYC*, *CCND1*, and *CTNNB1* in gastric cancer specimens (295 patients) from TCGA database^4^ (https://cancergenome.nih.gov/). **(B)** Positive association between the expression of EphA2 and of downstream target genes of the Wnt/β-catenin pathway in gastric cancer tissues in a correlation analysis with TCGA data. **(C)** Differences between EphA2^high^ and EphA2^low^ gastric cancer specimens revealed by GSEA of TCGA database. Wnt signaling target gene sets (MYC-targets), were enriched in the EphA2high gastric cancer specimens.

**
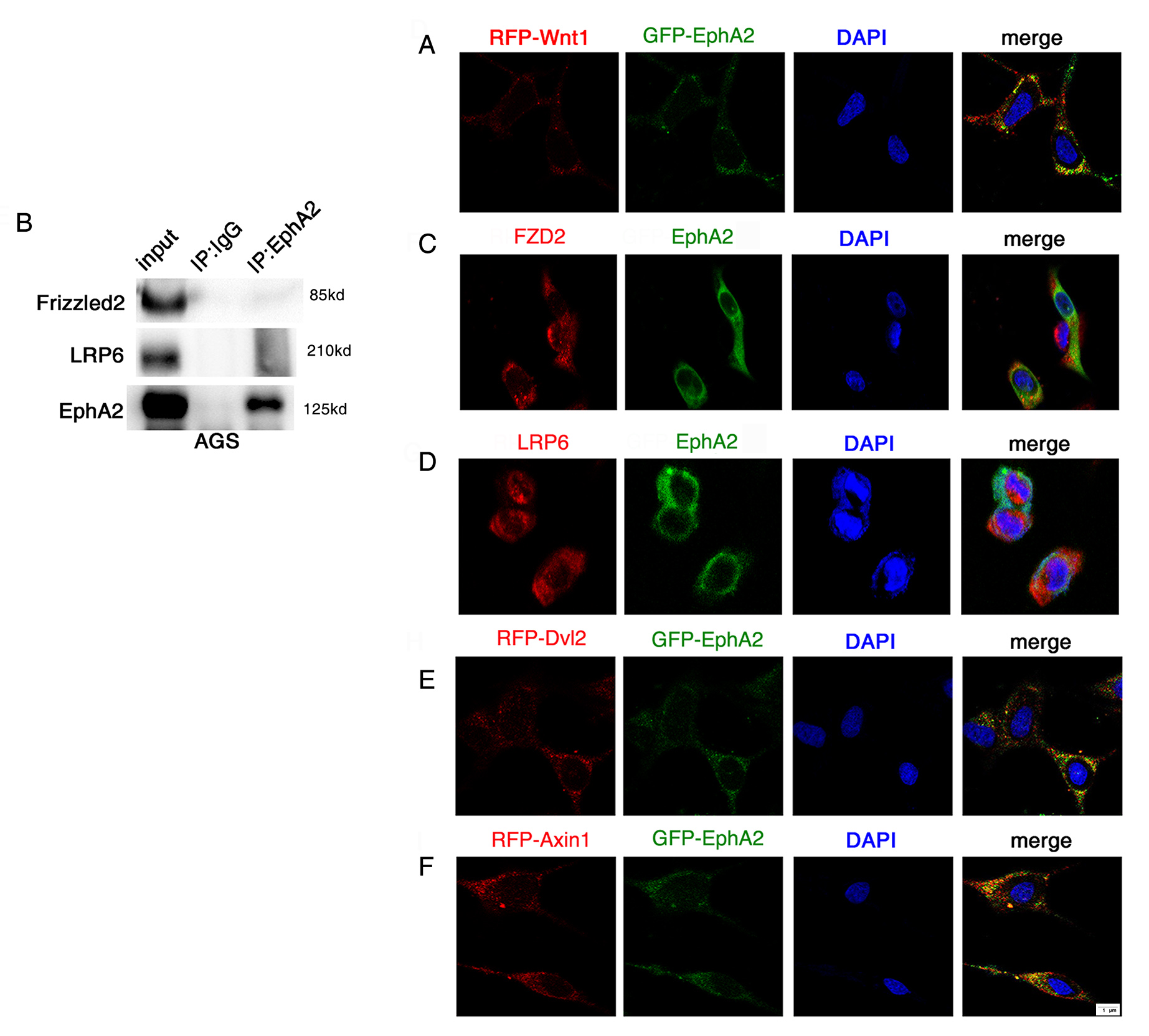
**

**Figure S3 (A)** Co-localization of GFP-EphA2 and RFP-Wnt1 after co-expression in HEK293 cells for 48 h as assessed with immunofluorescence staining. **(B)** Interaction of endogenous EphA2 with Frizzled2and LRP6 in AGS cells. **(C, D)** Co-localization of endogenous EphA2 and Frizzled2 **(C)**, LRP6 **(D)** in AGS cells as assessed with immunofluorescence staining. **(E, F)** Immunofluorescence staining to examine the co-localization in HEK293 cells of **(E)** EphA2 and Dvl2 after co-expression of GFP-EphA2 and RFP-Dvl2, and **(F)** EphA2 and Axin1 after co-expression of GFP-EphA2 and RFP-Axin1.


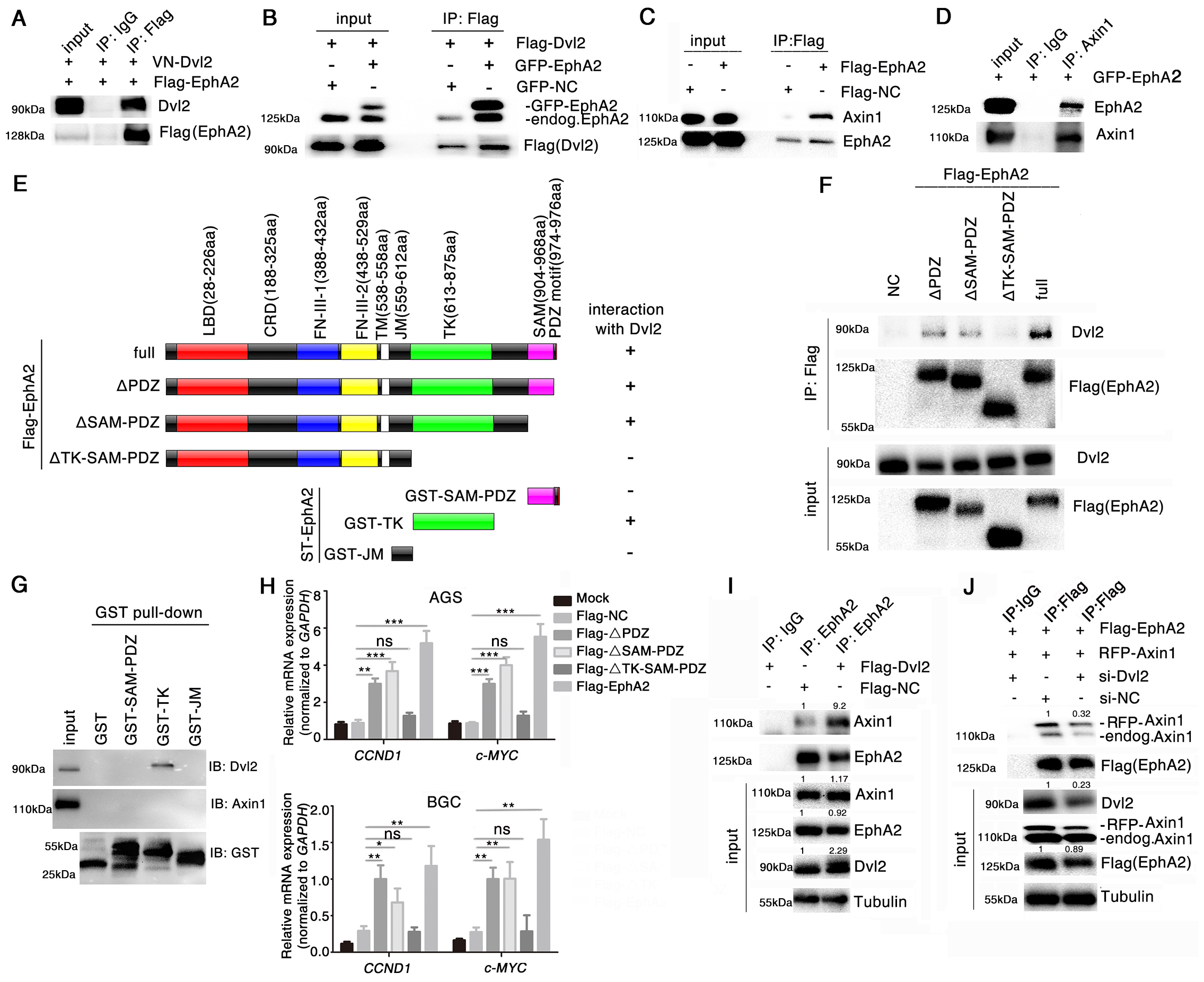


**Figure S4. (A-D)** Exogenous interaction between EphA2 with Dvl2 or Axin1 after transient expression of the indicated expression plasmids in HEK293 cells for 48 h. Western blotting of cell lysates subjected to co-IP with **(A, B, C)** anti-Flag or **(D)** anti-Axin1. **(E)** Schematic diagram of a series of N-terminal Flag-tagged and N-terminal GST-tagged EphA2 mutant fusion proteins and their interactions with Dvl2. **(F)** Interaction between endogenous Dvl2 and the N-terminal Flag-tagged EphA2 mutant proteins. AGS cells were transiently transfected with a series of N-terminal Flag-tagged EphA2 mutants followed by co-IP with anti-Flag. **(G)** GST pull-down assays reveal that the tyrosine kinase domain of EphA2 could pull down Dvl2 but not Axin1. (**H**) Expression of *CCND1* and *c-MYC* in AGS and BGC cells after transfected with the indicated plasmids as assessed with real-time qPCR. **(I)** Interaction between endogenous EphA2 and Axin1 in BGC823 cells that were transfected with the indicated plasmids and analyzed by co-IP using anti-EphA2. **(J)** Interaction between exogenous EphA2 and Axin1 in BGC823 cells transfected with the indicated plasmids and si-Dvl2, analyzed by co-IP using anti-Flag. Relative accumulations of proteins in different groups compared with the negative control group are indicated. Significant differences were determined with the Student’s *t*-test. **P* < 0.05, ***P* < 0.01, ****P* < 0.001 compared with control group.


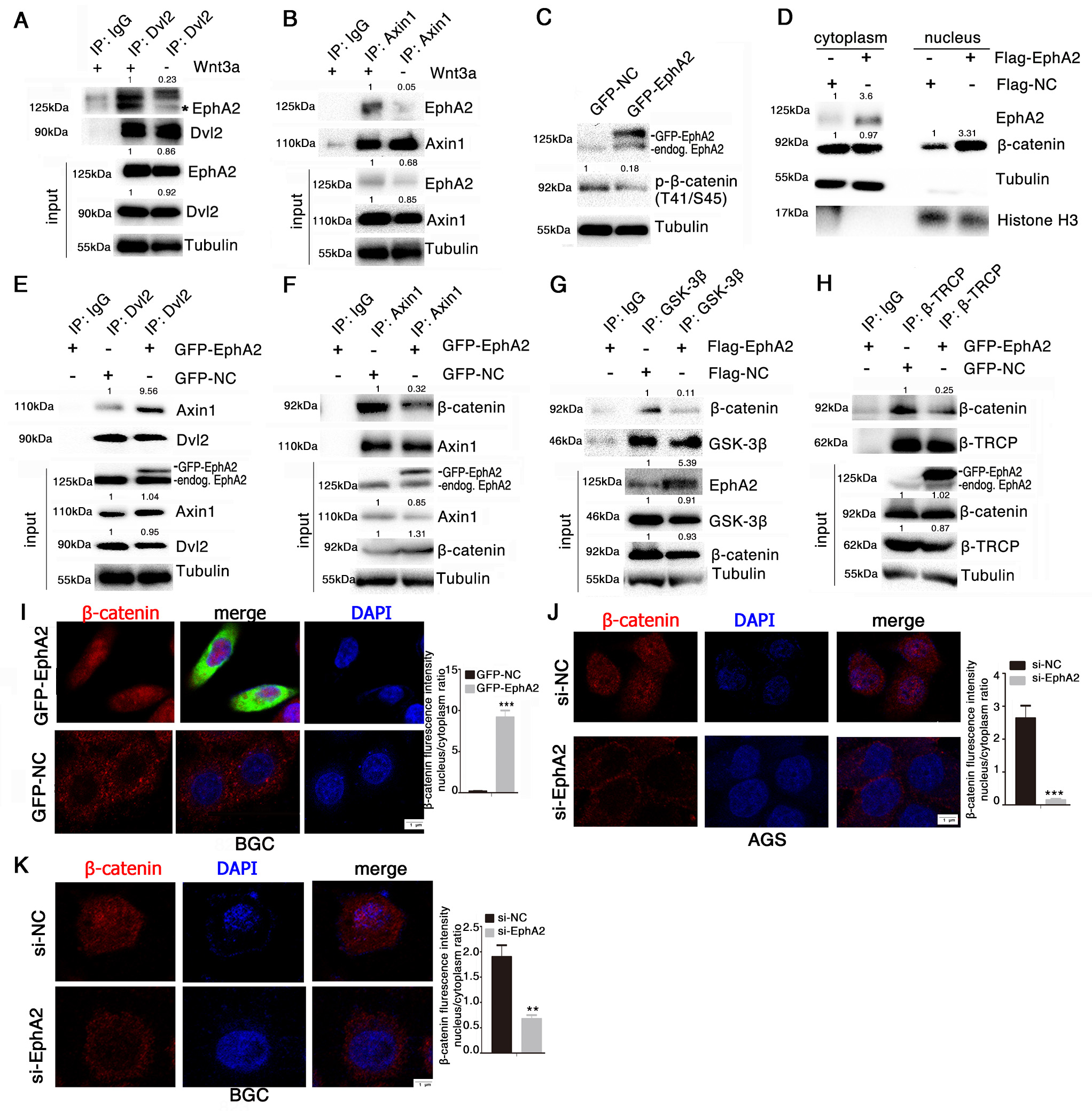


**Figure S5. (A,B)** Interaction of endogenous EphA2 with Dvl2 (A) or Axin1 (B) with or without Wnt3a stimulation in BGC823 cells. **(C)** Phosphorylation of β-catenin at residues Thr41/Ser45 after transfection of BGC823 cells with GFP-EphA2 for 48 h. **(D)** Western blotting reveals β-catenin and EphA2 distribution in the nuclear and cytoplasmic fractions at 48 h post-transfection with an EphA2 or negative control expression vector in BGC823 cells. **(E–H)** EphA2 expression plasmids transiently expressed in AGS cells for 48 h. Cell lysates were subjected to co-IP followed by western blotting for **(E)** Dvl2 and Axin1 revealed by anti-Dvl2, **(F)** Axin1 and β -catenin revealed by anti-Axin1, **(G)** GSK-3β and β-catenin revealed by anti-GSK-3β, and **(H)** β-catenin and β-TRCP revealed by anti-β-TRCP. **(I)** Effect of EphA2 overexpression on the subcellular localization of β-catenin monitored by immunofluorescence in BGC823 cells. **(J, K)** Effect of silence EphA2 on the subcellular localization of β-catenin monitored by immunofluorescence in AGS cells**(J)** or BGC823 cells**(K)**. Relative accumulations of proteins in different groups compared with the negative control group are indicated. Significant differences were determined with the Student’s *t*-test. ***P* < 0.01, ****P* < 0.001 compared with control group.


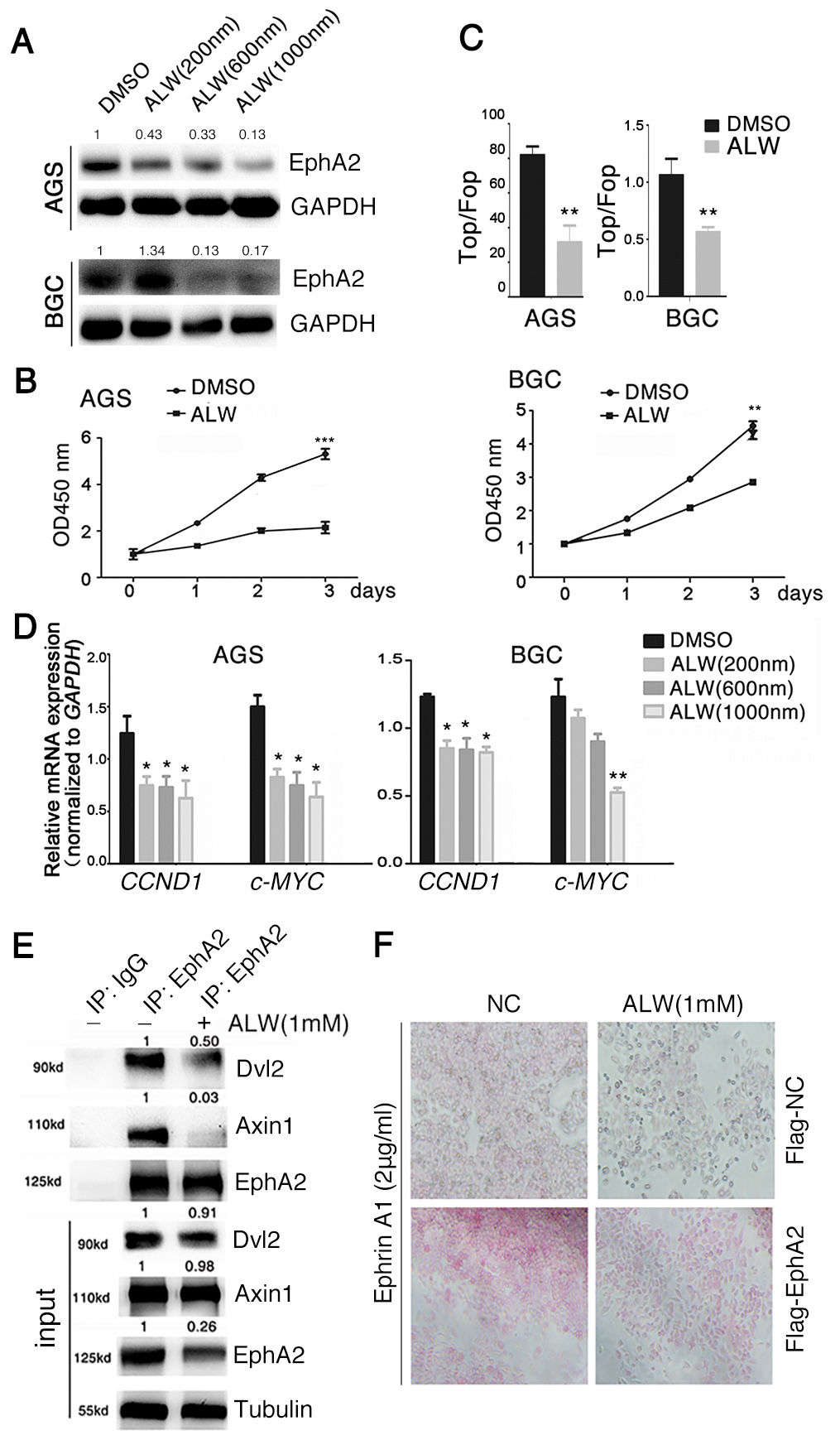


**Figure S6. (A)** ALW treatment decreases EphA2 protein levels in cells in a dose-dependent manner. **(B)** ALW treatment decreases cell proliferation in the CCK8 assay. **(C)** ALW treatment decreases TOP-flash/FOP-flash luciferase reporter activities. **(D)** ALW treatments decrease mRNA expression of *c-MYC* and *CCND1*. **(E)** Interaction of endogenous EphA2 with Dvl2 /Axin1 with or without ALW stimulation in AGS cells. **(F)** Flag-EphA2 and Flag-NC was transfected into HEK293 cells for 48h and incubated with 2μg/ml EphrinA1 for an additional 3h. EphrinA1 binds EphA2 at the cell surface. Staining was performed as described in the Methods. Relative accumulations of proteins in different groups compared with the negative control group are indicated. Significant differences were determined with the Student’s *t*-test. **P* < 0.05, ***P* < 0.01, ****P* < 0.001 compared with control group.


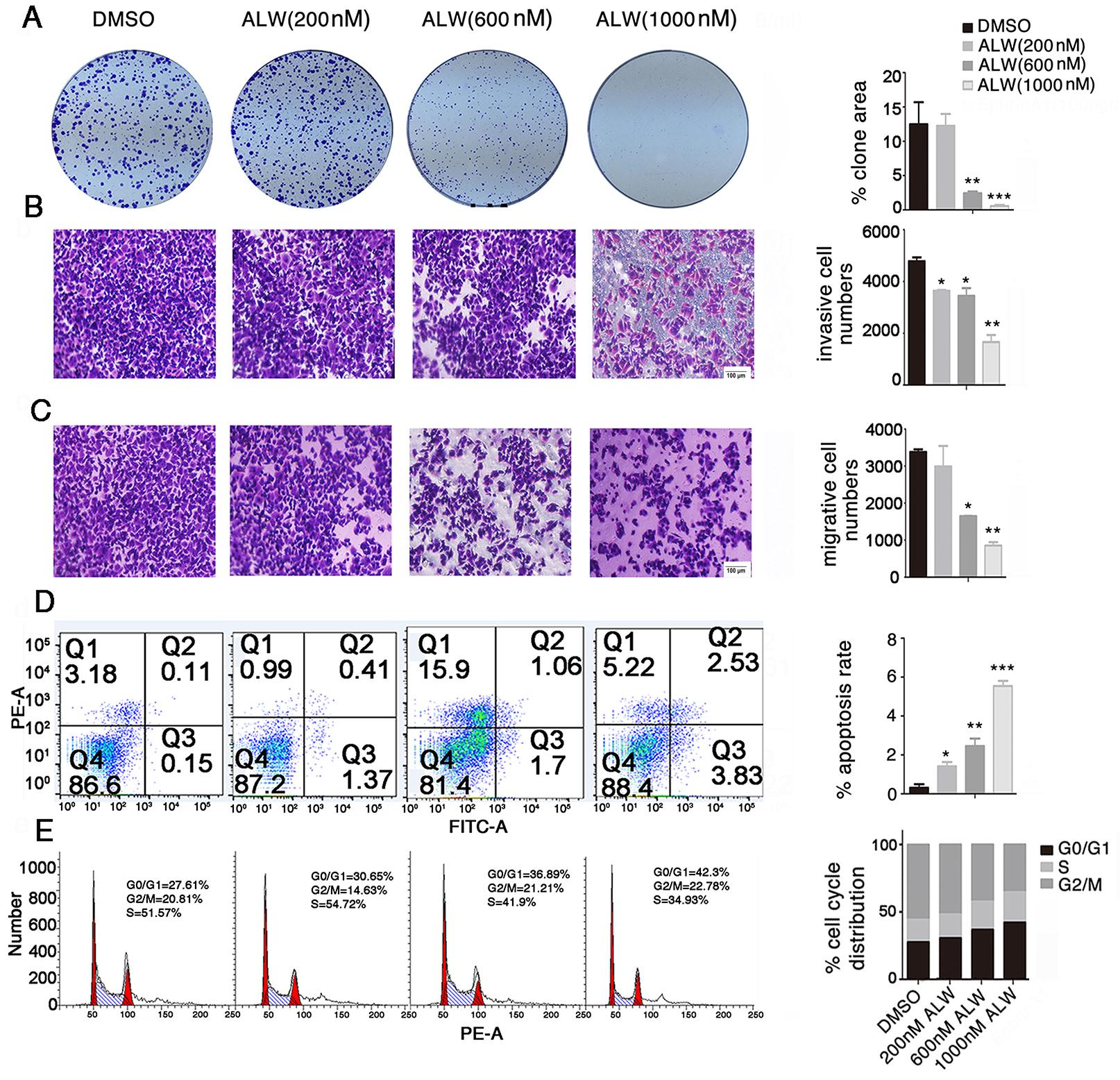


**Figure S7.** ALW suppresses gastric cancer tumorigenesis in BGC823 cells *in vitro* in a dose-dependent manner. Cell parameters were determined with **(A)** cell clone-formation assay, **(B, C)** transwell Matrigel assays for **(B)** cell invasion and **(C)** migration assay, **(D)** apoptosis analysis, and **(E)** cell-cycle analysis. Significant differences were determined with the Student’s *t*-test. **P* < 0.05, ***P* < 0.01, ****P* < 0.001 compared with control group.


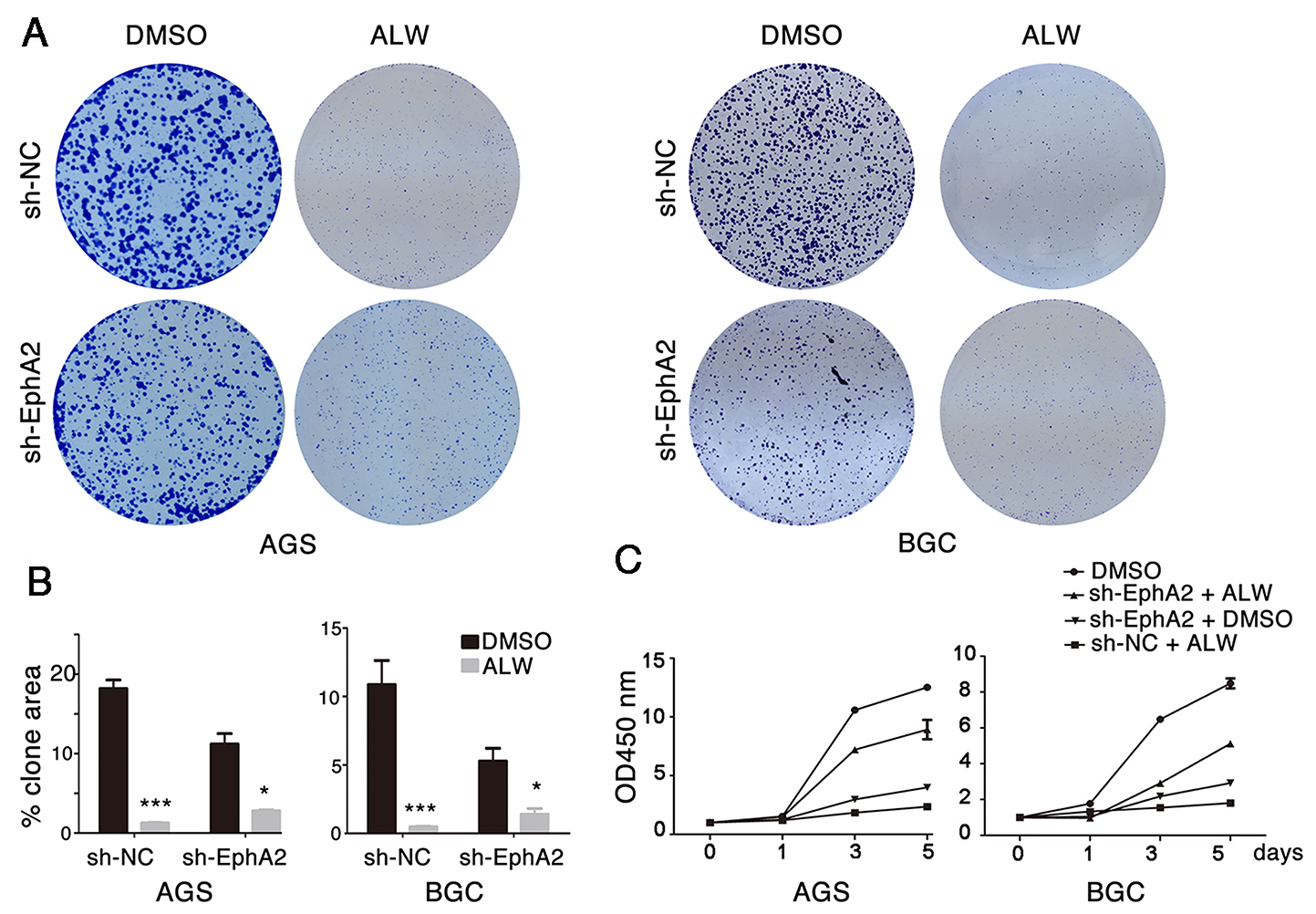


**Figure S8.** Inhibition of endogenous EphA2 in AGS and BGC823 cells treated with sh-EphA2. Cells were treated with ALW, and cell parameters were determined with **(A)** cell clone-formation assays, **(B)** clone area quantification, and **(C)** CCK8. **P* < 0.05, ****P* < 0.001 compared with control group.

**
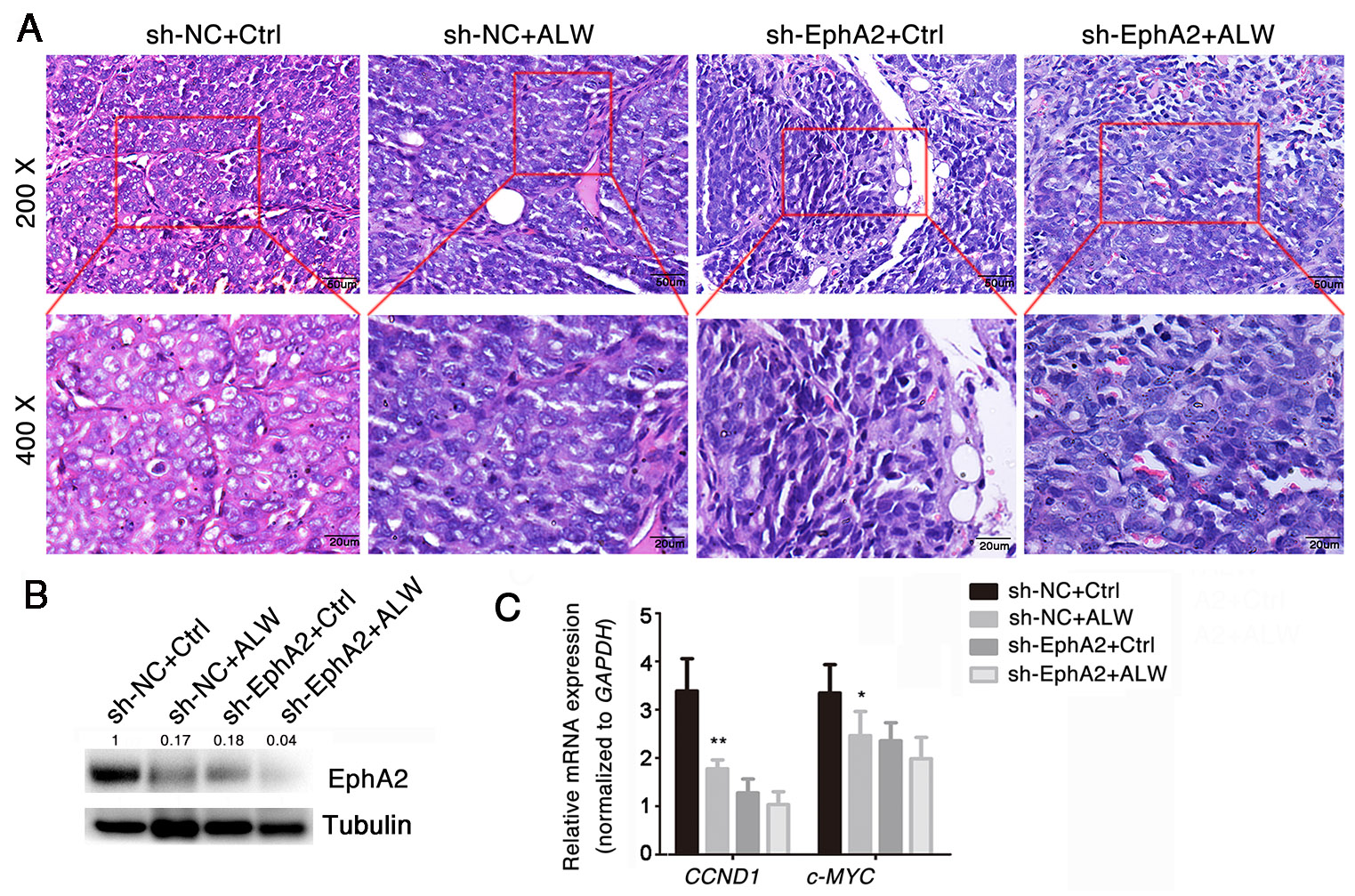
**

**Supplementary Figure S9 A-C**

**Figure S9.** sh-NC or sh-EphA2 AGS cells (5×10^6^) were injected subcutaneously into the dorsal flanks of nude mice. Tumors were allowed to grow to ~100 mm^3^ before administration of 20 mg/kg ALW (or control vehicle alone) via intraperitoneal injection once daily. **(A)** Representative images of hematoxylin and eosin–stained mouse tumor sections. Top row: 200 × magnification; bottom row: boxed regions from the top row shown at 400× magnification. **(B)** EphA2 level in mouse tumor tissues assayed by western blotting. **(C)** Expression of *CCND1*, and *c-MYC* in mouse tumor tissues as assessed with real-time qPCR. **(D)** Apoptosis in different mouse tumor tissues assessed with TUNEL staining. **(E)** Cell proliferation assessed with Ki-67 staining. Tumor microvessel density assessed with anti-CD31 antibody staining. **(F)** EphA2, β-catenin, and c-MYC levels in mouse tumor tissues assessed with immunohistochemistry. Relative accumulations of proteins in different groups compared with the negative control group are indicated. Significant differences were determined with the Student’s *t*-test. **P* < 0.05, ***P* < 0.01, ****P* < 0.001 compared with control group.

**
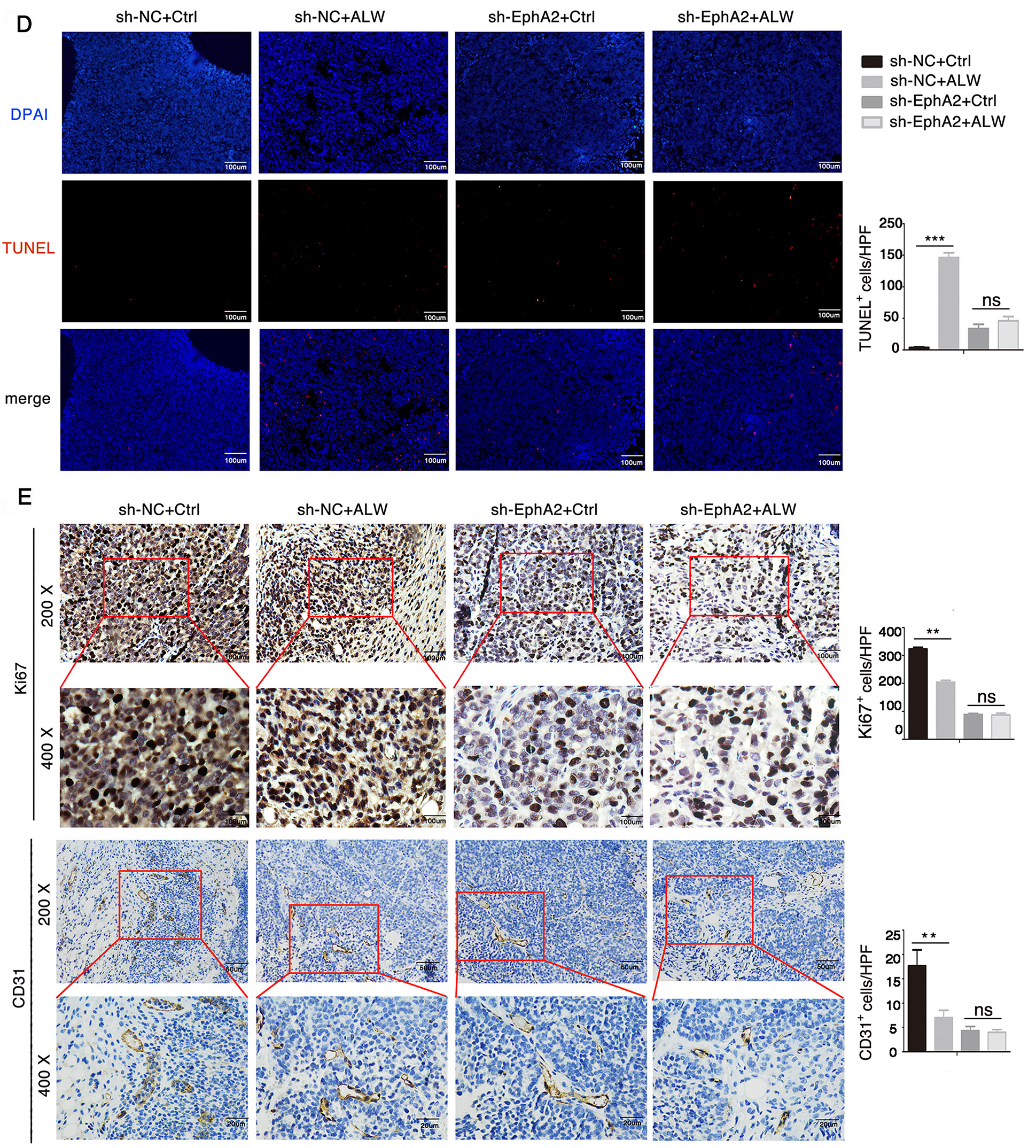
**

**Supplementary Figure S9 D-E**

**
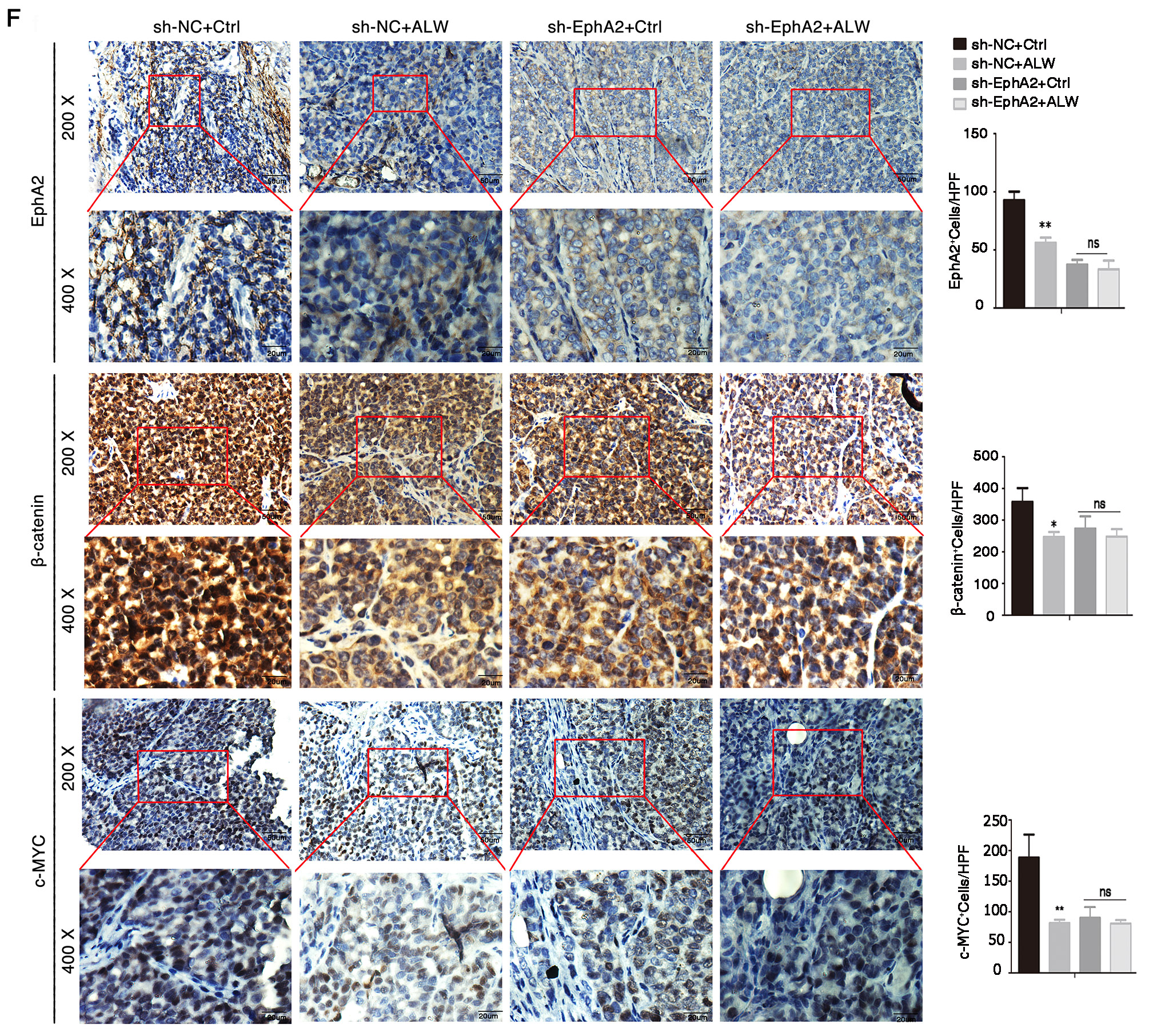
**

**Supplementary Figure S9 F**

**References**

1 Cho, J. Y. *et al.* Gene expression signature-based prognostic risk score in gastric cancer. *Clin Cancer Res* **17**, 1850-1857, doi:10.1158/1078-0432.CCR-10-2180 (2011).

2 D'Errico, M. *et al.* Genome-wide expression profile of sporadic gastric cancers with microsatellite instability. *Eur J Cancer* **45**, 461-469, doi:10.1016/j.ejca.2008.10.032 (2009).

3 Wang, Q. *et al.* Upregulated INHBA expression is associated with poor survival in gastric cancer. *Med Oncol* **29**, 77-83, doi:10.1007/s12032-010-9766-y (2012).

4 Cancer Genome Atlas Research, N. Comprehensive molecular characterization of gastric adenocarcinoma. *Nature* **513**, 202-209, doi:10.1038/nature13480 (2014).
